# Comparative Genome Analysis of the High Pathogenicity *Salmonella* Typhimurium Strain UK-1

**DOI:** 10.1371/journal.pone.0040645

**Published:** 2012-07-06

**Authors:** Yingqin Luo, Qingke Kong, Jiseon Yang, Arindam Mitra, Greg Golden, Soo-Young Wanda, Kenneth L. Roland, Roderick V. Jensen, Peter B. Ernst, Roy Curtiss

**Affiliations:** 1 Center for Infectious Diseases and Vaccinology, The Biodesign Institute, Arizona State University, Tempe, Arizona, United States of America; 2 Center for Evolutionary Medicine and Informatics, The Biodesign Institute, Arizona State University, Tempe, Arizona, United States of America; 3 Department of Biological Sciences, Virginia Polytechnic Institute and State University, Blacksburg, Virginia, United States of America; 4 Department of Medicine, Division of Gastroenterology and Hepatology, University of Virginia, Charlottesville, Virginia, United States of America; Universite de la Mediterranee, France

## Abstract

*Salmonella enterica* serovar Typhimurium, a gram-negative facultative rod-shaped bacterium causing salmonellosis and foodborne disease, is one of the most common isolated *Salmonella* serovars in both developed and developing nations. Several *S.* Typhimurium genomes have been completed and many more genome-sequencing projects are underway. Comparative genome analysis of the multiple strains leads to a better understanding of the evolution of *S.* Typhimurium and its pathogenesis. *S.* Typhimurium strain UK-1 (belongs to phage type 1) is highly virulent when orally administered to mice and chickens and efficiently colonizes lymphoid tissues of these species. These characteristics make this strain a good choice for use in vaccine development. In fact, UK-1 has been used as the parent strain for a number of nonrecombinant and recombinant vaccine strains, including several commercial vaccines for poultry. In this study, we conducted a thorough comparative genome analysis of the UK-1 strain with other *S.* Typhimurium strains and examined the phenotypic impact of several genomic differences. Whole genomic comparison highlights an extremely close relationship between the UK-1 strain and other *S*. Typhimurium strains; however, many interesting genetic and genomic variations specific to UK-1 were explored. In particular, the deletion of a UK-1-specific gene that is highly similar to the gene encoding the T3SS effector protein NleC exhibited a significant decrease in oral virulence in BALB/c mice. The complete genetic complements in UK-1, especially those elements that contribute to virulence or aid in determining the diversity within bacterial species, provide key information in evaluating the functional characterization of important genetic determinants and for development of vaccines.

## Introduction

Members of the bacterial genus *Salmonella* are among the major pathogens that cause infections in humans and almost all known animals. *Salmonella enterica* serovar Typhimurium is a principal cause of food-related illness (16% of salmonellosis infections a year in the United States) [1]. Most nontyphoidal salmonellae (NTS) *Salmonella* infections among healthy adults are associated with gastroenteritis that resolves without treatment and associated with case fatality rate <1% [2]. Recently, invasive NTS have been associated with life-threatening systemic infections in sub-Saharan Africa and in susceptible populations, such as in adults with advanced HIV disease, and susceptible children [3], [4].

*S.* Typhimurium is an invasive enteric pathogen that is remarkably adaptable to diverse hosts including humans, poultry, rodents, cattle, sheep and horses. More than 200 different *S.* Typhimurium strains have been identified, which are principally adapted to niches in the environment and the intestines of different animal species [5]. Although the genome content of *S.* Typhimurium strains is extremely similar [6], different combinations of fitness factor-encoding mobile genetic elements and phages have been observed [7]. *S.* Typhimurium has been used extensively in the investigation of *Salmonella* pathogenicity and for recombinant vaccine development [8], [9], [10].

*S.* Typhimurium strain UK-1, a phage type 1 strain, is a chicken-passaged isolate of a highly virulent *S.* Typhimurium strain originally isolated from an infected horse in 1991 [9]. UK-1 is not only highly invasive and virulent for chickens and mice, but is also capable of lethal infections in calves, pigs and horses [11], [12]. Because of the high virulence of UK-1, attenuated derivatives of the UK-1 strain are expected to induce a higher level of protective immunity after oral administration than the attenuated derivatives of less virulent *S.* Typhimurium strains [12]. For example, in one study, an attenuated UK-1 derivative was shown to elicit higher levels of serum IgG to a heterologous antigen than a similarly attenuated derivative of strain SR-11 [13]. UK-1 has been extensively used in our laboratory for virulence and colonization studies in chickens and mice for over twenty years. UK-1 strain χ3761 was the parent strain from which the licensed vaccines for broilers and pullets, Megan®Vac and Megan®Egg, respectively, were derived [9], [14], [15], [16], [17] and attenuated derivatives have been evaluated as vaccines for calves [18], horses [19], and dogs [20]. In recent years this strain has been used as the foundation for developing recombinant vaccines [21], [22].

The pathogenesis of *S. enterica* has been extensively studied [23], [24], [25], [26]. The rapid increase of genomic sequence data has revolutionized the study of bacterial pathogens and led to many improvements in vaccine design. The availability of more genome sequences has lead to the discovery of additional genes and has fueled the new field of comparative genomics [27]. Comparative genome analysis is providing details on gene function and gene/genome evolution, leading to a better understanding of bacterial evolution and pathogenesis [28], [29]. Even though genome sequences of several *S.* Typhimurium strains such as LT2, 14028s, D23580, and SL1344 have been available in public databases [6], [30], [31], additional *S.* Typhimurium sequences would be a valuable resource for improving our understanding of the biology of this species [32], [33]. More importantly, comparison of multiple genomes in a particular species is a valuable aid for determining key differences between strains. These differences represent a genetic potential for each species that may not have been explored previously, and may be important for predicting emergence of drug resistance and new virulent forms of pathogens [34]. With genome-wide screening across multiple sequenced genomes, one could predict the genes that are linked to drug resistance or virulence, and identify vaccine candidates or antimicrobial targets [35], [36]. In addition, not all virulence factors in pathogens increase the virulence of all strains; i.e. virulence factors in one strain may be dispensable for virulence or may actually decrease virulence when present in another strain. For example, the adhesion protein YadA is a virulence factor for *Yersinia enterocolitica,* but expression of *Y. pseudotuberculosis yadA* in *Y. pestis* reduces its virulence [37], [38]. In the post-genomic era, whole genome analysis of multiple strains within one species has become an important and necessary approach for understanding bacterial species, in particular, pathogens with diverse virulence factors.

*S.* Typhimurium UK-1 is the main platform for vaccine study in our lab. An approach based on whole-genome comparison was applied to determine the complete genetic complements of known *S.* Typhimurium strains, which may determine the diversity of species and contribute to virulence of strains.

## Results and Discussion

### Virulence of the *S.* Typhimurium Strains

*S.* Typhimurium strain D23580 is a human host-adapted strain dominant in Africa [31], while UK-1, 14028s, and SL1344 are non-host-adapted strains [9], [39], [40], [41]. We examined the virulence of the three non-host-adapted strains by measuring the median lethal dose (LD_50_). *S*. Typhimurium strain UK-1, which has an oral LD_50_ of 2.5×10^4^ CFU with a lower limit of 6.6×10^3^ CFU and an upper limit of 9.1×10^4^ CFU (95% confidence level), was the most virulent (Table 1). Strains SL1344 and 14028s had LD_50_s that were 3-fold and 4-fold higher, respectively, than UK-1, although these differences were not statistically significant, since the confidence interval (CI) values for all three strains overlapped.

**Table 1:** Virulence of wild-type *S.* Typhimurium strains for orally inoculated BALB/c mice.

Strain	LD_50_ (CFU)	Lower bound 95%	Upper bound 95%
UK-1	2.5×10^4^	6.6×10^3^	9.1×10^4^
14028S	9.6×10^4^	4.2×10^4^	2.2×10^5^
SL1344	7.8×10^4^	3.5×10^4^	1.8×10^5^

### General Genomics Features of UK-1 and Other *S.* Typhimurium Strains

**Figure 1:**
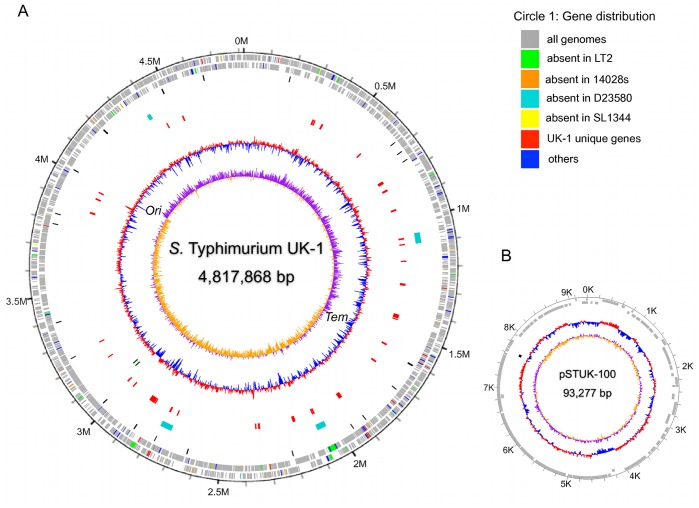
Genome atlas of *Salmonella enterica* serovar Typhimurium UK-1. (A) The chromosome. Base pairs are indicated outside the outer circle. The circles represent the following (from outside to inside): Circle 1 shows the distribution of predicted ORFs in the leading and lagging strands (see details in the color legend for Circle 1). Circle 2 shows the UK-1 pseudogenes (black, single circle). Circle 3 shows the phage regions in UK-1 (Cyan, single circle). Circle 4 shows the genomic islands predicted by IslandViewer [98] (red, single circle). Circle 5 displays the GC content of the genome (red: high GC content, purple: low GC content). Circle 6 displays GC skew ([G+C]/[G−C]) plot. (B) The UK-1 plasmid pSTUK-100 genome. Base pairs are indicated outside the outer circle. From outside to inside: genes predicted in the plasmid genome (two circles; all ORFs are shown in grey since there were no unique genes found in the pSTUK-100 genome.), pseudogene(s) identified in pSTUK-100 (black, single circle), GC content of the plasmid genome (red: high GC content, purple: low GC content), and GC Skew Plot. For the GC content and GC skew analysis, we applied a sliding window of 1,000 bp with an overlap of 500 bp. The atlas was created using GenomeViz software [99].

**Figure 2:**
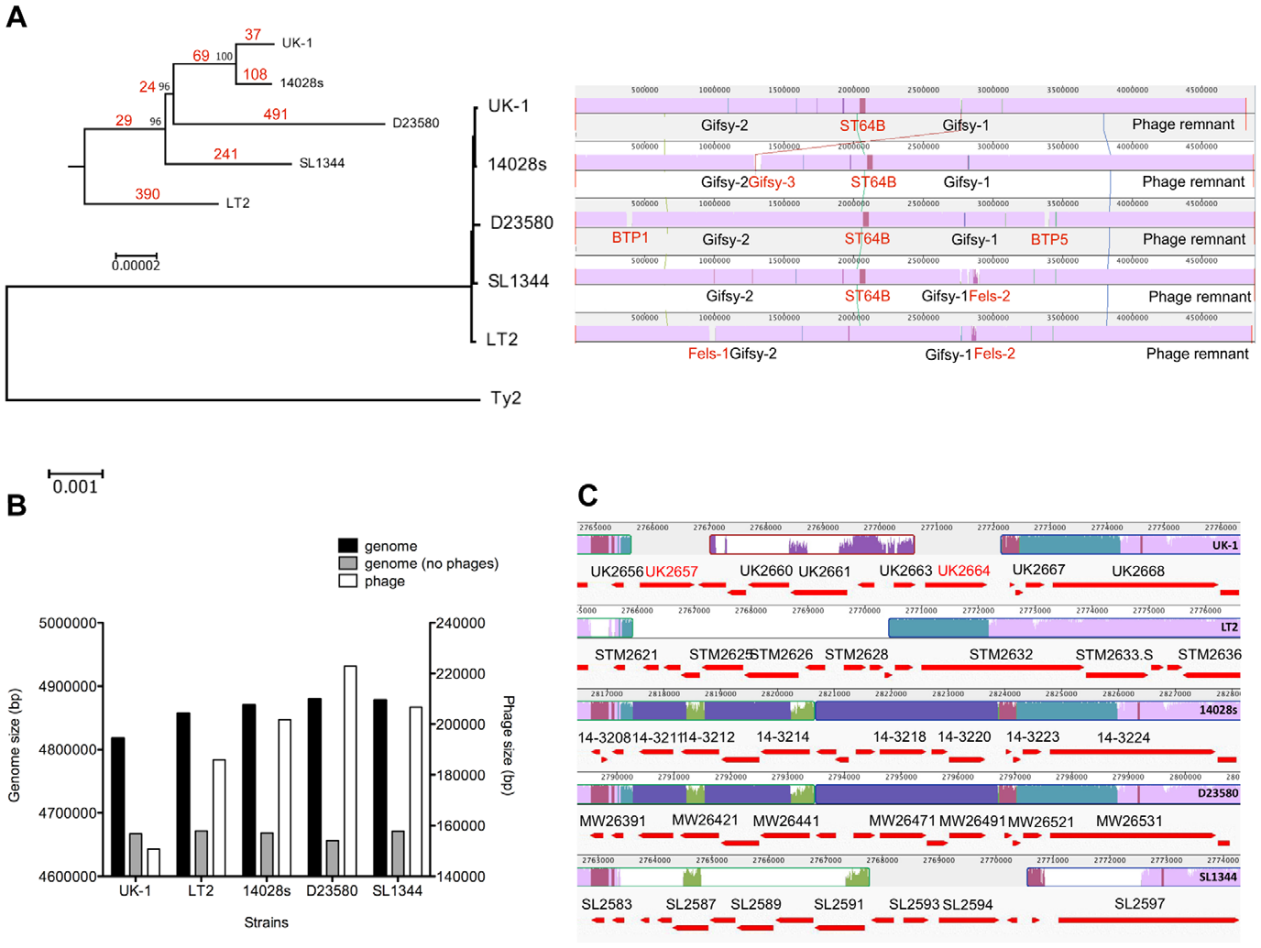
Phylogenetic relationship of the five *S.* Typhimurium strains. (A) The phylogenetic tree was inferred with ML method based on the conserved genomic sequences. The *S.* Typhimurium strains are rooted to *S.* Typhi Ty2. The upper-left subtree shows the phylogenetic relationship of the five strains in a smaller scale. The relationship was supported by the bootstrapping values shown on the subtree. The distance (marked in red) based on the number of SNPs was also presented on the phylogenetic tree. The right panel shows the complete genome alignment of the five strains generated in MAUVE [93]. The regions conserved among all genomes are colored in purple and the regions conserved among subsets of the genomes are colored differently. If the areas contain sequence elements not aligned, those are marked in white. Regions that are not colored indicate no detectable homology among the five genomes in MAUVE. The distinguished phages and phage remnants are marked on the alignment (black: detected among all of the five strains, red: detected in a subset of strains). (B) Comparison of the lengths of genomes, phages, and genomes excluding phage regions among the five *S.* Typhimurium strains. Length of phages is displayed on the second Y-axis due to the relatively small value of phages in contrast to the whole genome size. (C) Alignment of the UK-1 Gifsy-1 sequence segment harboring the two UK-1 unique genes with sequences from the other four *S.* Typhimurium strains. The sequence alignments were generated in MAUVE. The color scheme used for the alignment is described in Fig. 2A. The predicted genes in these regions are shown with red solid arrays. Each gene name is indicated with the strain name (UK indicates UK-1, STM indicates LT2, 14- indicates 14028s, MW indicates D23580, and SL indicates SL1344) followed by its locus number obtained from each of the annotation files. The two UK-1 unique genes are marked in red in the UK-1 genome.

The complete genome sequence of the UK-1 strain has been determined and annotated by our laboratory [33]. The general genomic features of UK-1 are represented in Fig. 1. The replication origin and terminus of UK-1, predicted by comparison with LT2 and confirmed by GC-skew [42], are near 4,004,924 bp and 1,503,568 bp, respectively. Comparison of the five genomes shows a high degree of similarity and gene synteny of genome core regions, including many of the *Salmonella* genetic islands (Fig. 2A and Fig. S1). Indeed, this comparative analysis highlights an extremely close relationship between UK-1 and LT2, 14028s, D23580, and SL1344. Of course it is the differences that we are most interested in and our comparison of the five strains discovered many features, including insertions, deletions, mutations and pseudogenes, which are related to genes with significant functions.

### Phages in the *S*. Typhimurium Strains

The *S.* Typhimurium strains analyzed so far carry between two and six prophages [7]. One of the differentiating features was a distinct repertoire of prophage-like elements in UK-1 (Fig. 2A). Of the four prophages found in UK-1, none were unique to UK-1. This is in contrast to the other strains, each of which contained prophages specific only to that strain and not occurring in the others (i.e., Fels-1 and Fels-2 specific to LT2, Gifsy-3 specific to 14028s, two prophages designated BTP1 and BTP5 specific to D23580, and a Fels-2-like prophage specific to SL1344; Fig. 2A). Interestingly, while UK-1 has four prophages, its genome carries the fewest number of bp corresponding to phage-specific sequences among the five S. Typhimurium strains (Fig. 2B).

Among the detected prophages, Gifsy-1, Gifsy-2, and a phage-like element were found in all five strains. A region of 11.6 kb inside Gifsy-1 is not conserved between the five *S.* Typhimurium genomes (Fig. 2C). It is one of the main polymorphic regions observed among the sequences of the five strains. In this region, a 3.5 kb segment was inserted into the UK-1 Gifsy-1 and shows distant homology to a segment of Gifsy-3 in 14028s. Another 6.4 kb segment was observed in 14028s and D23580, but has been replaced in UK-1, LT2, and SL1344 with other sequences unique to each of these strains. From the multiple genome alignments, it seems this region shows the typical gain and loss of sequences during genome evolution. The evolutionary relationship of this region is consistent with the phylogenetic tree, which proved to be a useful framework to investigate the recent evolution of phenotypic traits *S.* Typhimurium. Based on the phylogenetic tree, the ST64B-like phage, Gifsy-3, BTP1, and BTP5 are events of phage gain that occurred after divergence from the attenuated strain LT2. Fels-1 is missing in the virulent strains and SL1344 carries only remnants of Fels-2. Thus, it appears that Fels-1 and Fels-2 represent phage loss in the virulent strains. Alternatively, due to the important role of phages in horizontal gene transfer [7], it is possible that Fels-1 and Fels-2 were acquired by LT2 after divergence from the other lineages. It is estimated that these phage related events occurred less than 3,000 years ago [6].

### Specific Genes in UK-1

As observed previously in other *S.* Typhimurium genomes [6], [31], the genome content of UK-1 is highly similar to LT2, 14028s and other available *S.* Typhimurium genomes. A broader search through the whole genomes of LT2, 14028s, D23580 and SL1344, including both coding and noncoding regions, found only two genes that were unique to the UK-1 strain. Based on blast searches of the possible homologous genes in public databases, the two genes are related to the type III effector system and they are homologous to genes from the prophage Gifsy-3. The two UK-1 unique genes are located in prophage Gifsy-1, designated as STMUK_2657 and STMUK_2664 (red marked genes in Fig. 2C). STMUK_2657 is homologous to the gene encoding a non-LEE encoded type III effector NleC-like protein (BLASTP identity  = 73% and e-value <1e-173). STMUK_2664 is homologous to the gene coding for a regulatory phage protein CII (BLASTP identity  = 57% and e-value <4e-154). To determine whether these sequences play a role in virulence, we constructed deletion mutations and tested the virulence of the resulting ΔSTMUK_2657 and ΔSTMUK_2664 deletion strains compared to the UK-1 parent when orally administered to BALB/c mice (see detailed results in Table S1). The LD_50_ value of the ΔSTMUK_2664 strain was similar to that of the UK-1 parent, indicating there was no effect of the deletion on virulence (Table 2). In contrast, LD_50_ value of the ΔSTMUK_2657 deletion strain was 10-fold higher than the LD_50_ value of the UK-1 parent. This difference was significant because the confidence intervals did not overlap, thus indicating that the STMUK_2657 sequences enhance the virulence of UK-1 and therefore constitute a newly discovered virulence factor.

### Pseudogenes

Pseudogenes are commonly observed in *Salmonella* genomes. They are usually created by deletions or insertions that cause a frame shift or by a nonsense SNP resulting in a stop codon within coding regions. Previous studies have identified pseudogenes in LT2, 14028s and D23580 [6], [30], [31], but typically only a few *S. *Typhimurium strains were included in the analyses. For example, the pseudogenes in 14028s were determined by comparison to LT2 only. A more extensive comparison of the pseudogenes conducted using UK-1, LT2, 14028s, D23580 and SL1344 showed that many of the 14028s specific pseudogenes were also observed in UK-1 (Table S2), D23580 and SL1344. These genes include *ratB*, *lpfD*, *yacH*, STMUK_1876, STMUK_2665, and STMUK_1639. Two other genes, *ybeU* and STM1228, were degraded or deleted in UK-1, 14028s, and SL1344, but were present in D23580 and LT2. In addition, the *nupG*, *alkA*, STMUK_3244, STMUK_3243, and STMUK_0617 genes were degraded in UK-1 and 14028s but were present in LT2, D23580, and SL1344. In total, there were 22 pseudogenes detected in the UK-1 chromosome (see the full list of the pseudogenes in Table S2). The LT2 gene STM2911 was degraded in UK-1 due to a single ‘T’ deletion within the coding region 65 bp upstream of the 3′-end of the gene STMUK_2900. BLAST results indicated that STMUK_2900 probably encodes a membrane translocase similar to the *Escherichia coli emrB* gene that confers multidrug resistance. There are more pseudogenes in D23580 than in UK-1 and 14028s, most likely due to the genome degradation associated with its evolution into a host-adapted strain [31]. In addition, two SL1344 genes, designated STM1833 and STM1896 and corresponding to UK-1 pseudogenes STMUK_1806 and STMUK_1876, were identified as being important for survival and replication in macrophage-like cells or in the spleens of BALB/cJ mice by a microarray-based transposon tracking strategy [43].

### Polymorphism Sites

Multiple alignments comparing the UK-1 genome with the genomes of strains LT2, 14028s, D23580, and SL1344 were processed further to identify three types of genetic differences: (i) single nucleotide polymorphisms (SNP), (ii) inserted or deleted sequences (Indel) and (iii) variation number of tandem repeats (VNTR).

**Table 2:** Virulence comparison between the ΔSTMUK_2657 and ΔSTMUK_2664 mutants and the UK-1 parent strain for orally inoculated BALB/c mice.

Strain	LD_50_ (CFU)	Lower bound 95%	Upper bound 95%
UK-1 wt	1.5×10^4^	6.0×10^3^	3.8×10^4^
UK-1 ΔSTMUK_2657	5.7×10^5^	1.6×10^5^	2.1×10^6^
UK-1 ΔSTMUK_2664	2.0×10^4^	8.0×10^3^	4.7×10^4^

**Figure 3:**
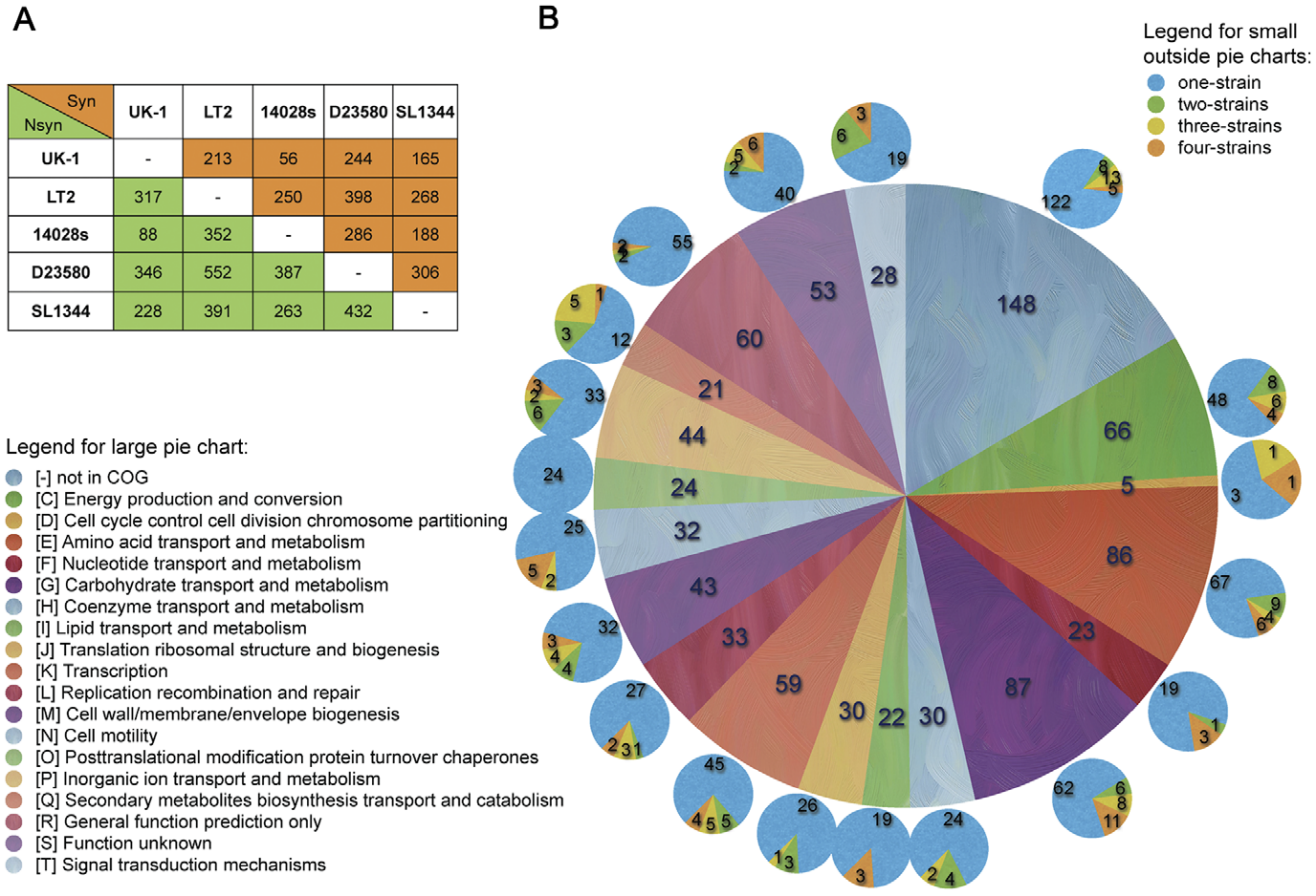
SNPs detected in the UK-1 strain with respected to the four *S.* Typhimurium genomes. (A) Pair-wise comparison of the synonymous and nonsynonymous SNPs among the five sequenced *S.* Typhimurium strains. (B) The distribution of UK-1 genes containing SNPs. The inner pie chart shows the number of genes carrying SNPs grouped by COG category [44]. For each group, the distribution of genes is shown in the outer pie charts, which describe the number of reference strains that contribute to the UK-1 SNPs. The legend of the inner pie chart is shown at the left of the pie charts. The legend of the outer pie charts is shown in the upper-right corner.

(i) SNP. SNPs were determined by pairwise comparison of genomic sequences from the five *S.* Typhimurium strains. Because of the transient nature of prophage presence and the fast decay of defective prophages in bacterial genomes, only SNPs detected outside of prophage sequences and repetitive regions were used in the analysis. The number of synonymous and nonsynonymous SNPs between each pair of strains are listed in the upper and lower triangular areas of Figure 3A. With reference to the other four genomes, 894 UK-1 genes carrying SNPs were detected and categorized based on the functional categories from the COG ontology database (Fig. 3B) [44]. 633 genes have well defined functions (379 for metabolism, 132 for cellular processes and signaling, and 122 for information storage and processing) and 261 are poorly characterized or have unknown function. For each COG category, the distribution of the UK-1 genes carrying SNPs was determined according to the number of reference genomes that provide the basis for the UK-1 SNPs. These are shown in Figure 3B as groups “one-strain”, “two-strains”, “three-strains” and “four-strains”. So for instance, group “one-strain” contains 702 genes identified as SNPs based on comparison to genes from only one of the other four genomes. Group “two-strains” contains 68 genes carrying SNPs with respect to genes from only two of the four genomes, and so forth. Most significantly, group “four-strains” contains 62 genes carrying SNPs with respect to genes from all four reference genomes. Thus, these 62 genes carry the UK-1-specific SNPs that are likely to be the most relevant for distinguishing UK-1 from the other four strains. Genes from the group “four-strains” occur in many functional COGs, suggesting that the SNPs could result in a variety of potential phenotypes. In order to understand the functional significance of the UK-1 SNPs, we referred to the known phenotypes of mutations in the polymeric genes produced by microarray-based experiments [43], [45], [46], [47]. Of the 894 genes carrying SNPs, 70 genes carrying nonsynonymous SNPs and 38 carrying synonymous SNPs were detected to be possible virulence-related factors in mice. The SNPs detected in the UK-1 strain with respect to the four other *S.* Typhimurium strains are listed in Table S3.

(ii) Indel. Indels can occur both in coding and non-coding sequences. Genomic regions with repetitive sequences make genome alignment more difficult since one repetitive region can be matched to several other regions. In addition, phage regions are highly divergent. For these reasons, we analyzed only indels detected outside of phages and repetitive regions. We detected sixteen deletions and 31 insertions in the UK-1 genome (see deletions in Table 3 and insertions Table S4). Indels DEL-06 and INS-04 were UK-1 specific, while the remaining indels were observed in one or more of the other genomes. DEL-06 is a deletion of twelve base pairs inside the UK-1 gene *nlpD* and INS-04 is an insertion of 18 bp in the UK-1 gene STMUK_2562 (corresponding to STM2530 in LT2), encoding a putative anaerobic dimethyl sulfoxide reductase. Four indels (DEL-04, INS-10, INS-13, and INS-20) occur in the genes STMUK_1666, *pckA* (STMUK_3486), *ybiP* (STMUK_0838), and STMUK_3000, respectively (Table 3 and S4). These four genes are important during infection of BALB/c mice based on microarray analyses [43], [45], [47].

**Table 3:** Deletions detected in the UK-1 strain by referring to the other four *S.* Typhimurium strains.

Id	Location	Reference strain	Configuration	Strand	Genes	Frame shift
DEL-01	184614	LT2	GATGATCT	−	*yacH*	YES
DEL-02	282141	LT2	GTAT	+	STMUK_0243	YES
DEL-03	314636	LT2	CAACAGGCGCTGGCG	+	STMUK_0276	NO
DEL-04	1748037	LT2	GC	−	STMUK_1666	YES
DEL-05	2979255, 2979296	LT2, D23580,and SL1344	A, ACGATAAAAAACTCTCTAT ATCCGCTCATAAAAAAAGG ATAGCTGAATATAAGTCTT TACTTAAACCGTAA	−	*avrA*	NO
DEL-06	3035962	LT2, 14028s, D23580,and SL1344	CTTGTTGCGGCG	−	*nlpD*	NO
DEL-07	3213439	LT2	ATGTCTGCGATGTCTGCG		Non-coding region	
DEL-08	3784996	LT2	ATTCTCAAAC		Non-coding region	
DEL-09	1185346	D23580	CGCTGGCGCTGG	+	*ycdZ*	NO
DEL-10	1190462	D23580	GG	+	STMUK_1115	
DEL-11	2038043	D23580	GATGGCGGT	+	*ompS*	NO
DEL-12	2331779	D23580	GTTGATGTA	+	*oafA*	Promoter
DEL-13	2875055	D23580	TTGCCGCGAT	−	STMUK_2752	YES
DEL-14	3728707	D23580	GGCATCGCCAGCGCC	−	STMUK_3580	NO
DEL-15	3769415	D23580	AA		Non-coding region	
DEL-16	69408	SL1344	TTA	+	*citC2*	NO

Indels may contribute to virulence and genome diversity when they are linked to genes with significant functions. For instance, *avrA*, encoding the virulence-associated effector protein AvrA, has been 3′-end truncated in both UK-1 and 14028s, compared with the other strains. AvrA is one of the 19 proteins within *Salmonella* pathogenicity island 1 (SPI-1) that are secreted by the type-3 secretion system (T3SS) within SPI-1 [48], [49], [50]. AvrA plays an anti-inflammatory role enhancing bacterial survival in the host [51]. We plan to explore the impact of the AvrA variation on virulence. In addition, DEL-12 is located within the promoter region (96 bp upstream) of *oafA*, which encodes O-antigen acetylase. It will be interesting to investigate the effect of these variations on *Salmonella* virulence.

Another interesting variation is a 12 bp deletion in *nlpD* [52]. *nlpD,* which encodes the lipoprotein NlpD, is located 62 bp upstream of the *rpoS* gene and 175 bp downstream of the *pcm* gene in UK-1. *nlpD* and *rpoS* constitute an operon [52] and *rpoS* is a virulence factor for *Salmonella* [53]. The major, growth phase-regulated *rpoS* promoter is located within the coding region of *nlpD* and a second promoter that does not appear to be regulated by growth phase lies upstream of *nlpD* [52], [54]. The location of the UK-1 deletion in *nlpD* falls outside of the *rpoS* promoter region [55], indicating that it is unlikely to influence *rpoS* expression. In *Yersinia pestis*, deletion of the *nlpD* gene sequence resulted in a drastic reduction in virulence for subcutaneous and airway routes of infection [56]. Comparison of the NlpD protein sequences from UK-1 and *Y. pestis* shows 60% identity between the two proteins. They contain conserved domains, indicating the possibility that the *S.* Typhimurium NlpD protein serves a similar function to the one in *Y. pestis*. Further functional studies are required to understand the full significance of these genes in *Salmonella*.

(iii) VNTR. A VNTR is defined as a tandem repeat that represents a single locus and shows length polymorphisms between individuals [57]. As another important type of genetic variation, VNTRs play a role in evolution, gene regulation, genome structure, and virulence [58], [59]. Furthermore, because it allows the bacterium to act swiftly based on deleterious environmental conditions [60], VNTRs have clear implications for virulence and antigenic variation [59]. All possible tandem repeats including short patterns (2–5 bp) and long patterns (>100 bp) were explored in the complete genome sequences of *S.* Typhimurium UK-1, LT2, 14028s, D23580, and SL1344. Altogether, 43 VNTRs with distinct patterns were detected among the five strains, 22 of which occurred in all five *S.* Typhimurium strains (Table S5). Of the 31 VNTRs observed in UK-1, 13 represent variations from other genomes (Table 4). These 13 VNTRs range from 6 to 184 bp, and 7 have repeat lengths of multiples of three. Three VNTRs are UK-1 specific, with lengths of 22, 105, and 53 bp and corresponding copy number 7.2, 2.5, and 2.9 respectively. VNTR-03, −13, −19, and −23 with unit length of 39, 6, 6, and 33 bp, respectively, carry different copy numbers among the five strain genomes (Table 4). The observations show that VNTRs are a major contribution to the diversity of *S*. Typhimurium genomes.

**Table 4:** VNTRs identified in the UK-1 genome that are not consistent in the five strains.

Id	Name	Repeat Configuration	UK-1 Locus	Strains				
				UK-1	LT2	14028s	D23580	SL1344
VNTR-02		[155 bp]	819953	2.6		2.6	2.6	2.6
VNTR-03	STTR7	[39 bp]	997189	7.5	8.1	7.5	7.5	7.5
VNTR-04		CAGCAGCCGGTAGCG CCGCAGCCACAGTAT	997444	2.3		2.3		
VNTR-05		GAAAACAGGGATAGTTATCCCC	1064337	3.6	3.6	3.6		3.6
VNTR-06		[184 bp]	1181777	2.4		2.4	2.4	2.4
VNTR-13	STTR6	GCAAGG	2730631	8.7	13.7	9.7	9.7	8.7
VNTR-14		[105 bp]	2768796	2.5				
VNTR-15		CTATCCCCGTTTTC[AG]GGGATAA	2768876	7.2				
VNTR-18		[121 bp]	3045681	2.2		2.2	2.2	2.2
VNTR-19	STTR5 orSal16	CACGAC	3151921	26.3	13.3	20.3	7.3	8.3
VNTR-22		ATGGCGGCAACGTC ACCCCGCCCGACG	3590943	3.3		3.3	3.3	3.3
VNTR-23	STTR3	[33 bp]	3591011	11.9	10.9	11.9	9.9	11.9
VNTR-28		[53 bp]	3917755	2.9				

These repeats, when present in genes with significant functions, should prove interesting for further study. VNTRs are believed to play a role in pathogen evasion of the host immune system [58], [59]. We identified many VNTRs in UK-1 that are related to genes within phage sequences or in genes with significant functions. For instance, VNTR-13 is located within phage Gifsy-1 between the virulence-associated gene *gogB*, encoding a leucine-rich repeat protein, and the gene STMUK_2617, encoding a transposase. VNTR-19 is located within *yohM*, a gene that codes for the nickel and cobalt efflux protein RcnA involved in nickel and cobalt resistance [61]. The variable copy numbers of VNTR-19 among the *S.* Typhimurium strains may be result differences in selective pressure imposed by these metals over the evolution of these strains [61]. VNTR-23, the most common VNTR locus among *S.* Typhimurium isolates, is located within *bigA*, a large gene encoding a surface-exposed adhesin protein, with 12, 11, 12, 10, and 12 copies in UK-1, LT2, 14028s, D23580, and SL1344, respectively.

### CRISPR

**Figure 4:**
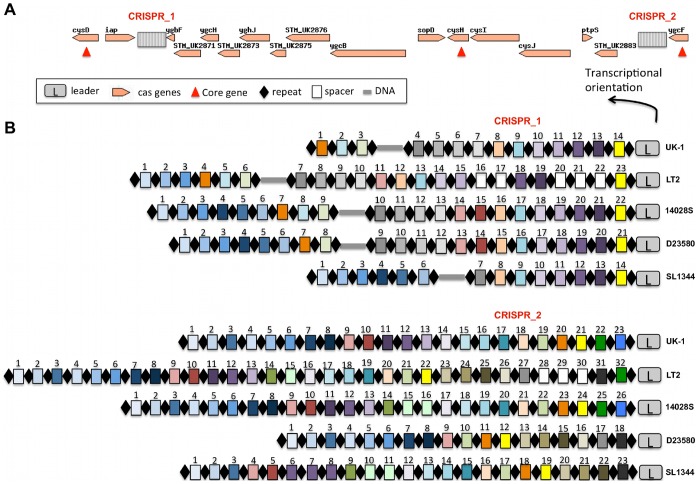
Two CRISPRs detected in the five S. Typhimurium strains. (A) Genetic map of the two CRISPR/*cas* systems present in *Salmonella* Typhimurium UK-1. 17 *cas* genes were detected around the two CRISPRs. Three core *cas* genes are noted with red triangles. (B) Overview of the two CRISPR loci in the five *S.* Typhimurium strains. The repeats are shown as dark diamonds. Spacers are shown as colored rectangles. In each CRISPR, spacers with identical sequence in the studied genomes are shown in the same color. The white rectangles indicate the strain specific spacers.

Clustered regularly interspaced short palindromic repeats (CRISPRs) are a distinctive feature of the prokaryotic genomes [62], [63]. CRISPRs, together with CRISPR associated sequence (CAS) proteins, have recently been discovered as a novel prokaryotic immune-like system involved in resistance to bacteriophage infection [64], [65], [66], [67]. This has been linked to the acquisition of CRISPR sequences from infecting phage. CRISPRs have hypervariable genetic loci due to their high diversity of spacers (interspaced regions between palindromic repeats). CRISPR_1 and CRISPR_2, previously analyzed in LT2 [68], were found in UK-1, 14028S, D23580 and SL1344 (Fig. 4A).

17 CRISPR-associated (*cas*) genes were located around CRISPR_1 and CRISPR_2 in all five *S.* Typhimurium strains. The palindromic repeats showed high similarities among the five strains, but the spacers were variable (Fig. 4B). For instance, in CRISPR_1, UK-1 lacks the first six spacers observed in 14028S, D23580 and SL1344. Additionally, five spacers in CRISPR_1 and three in CRISPR_2 were observed only in LT2. Several studies reported that many spacers frequently match to phage and other extrachromosomal elements [69], [70], [71]. However, we found that all spacers in CRISPR_1 and CRISPR_2 from all five strains were unique sequences with no homology to known phages or extrachromosomal elements. Interestingly, one spacer, spacer 17 in LT2 CRIPSR_1 matched with 100% identity to many eukaryotic sequences. Thus, in *S.* Typhimurium we found no sequence information to support a role for CRISPRs in phage immunity. Alternatively, some authors have suggested that non-identity-spacers might mediate the interaction between CRISPRs and bacteriophage [72], [73]. For instance, spacer 1 of *Pseudomonas aeruginosa* strain UCBPP-PA14 is not identical to any region of the phage DMS3 genome, but mediates DMS3-dependent loss of biofilm formation [72]. Removal or addition of particular spacers modified the phage-resistance phenotype of the cell [64]. The spacer diversity among the five *S.* Typhimurium strains indicates that the CRISPRs may play some interesting roles other than in phage immunity.

### Polymorphisms in the *Salmonella* Virulence Plasmid

The large virulence-associated plasmid present in UK-1, pSTUK-100, is closely related to the LT2 plasmid pSLT and similar plasmids from 14028s, D23580 and SL1344. The five plasmids are likely to have the same ancestor. Comparing the sequence of pSTUK-100 with the other four plasmids, several deletions were detected to be pSTUK-100 specific. For example, a 578 bp region in a putative adhesion protein gene defined in pSLT, was absent in pSTUK-100, but was present in other studied virulence plasmids (Fig. S2). This novel deletion in UK-1 might have an effect on virulence since there is evidence that deletion of genes can lead to enhanced virulence of pathogens [37], [74]. A 6 bp segment in *spvB* (GCCACC) was absent in pSTUK-100. *spvB* is a structural gene in the *spv* (*Salmonella* plasmid virulence) operon that is important for *Salmonella* virulence in mice [39], [75]. In addition, deletions in pSTUK-100 were also detected in other *S.* Typhimurium plasmids, including the 81 nucleotide deletion in *traD* observed in the plasmids of UK-1, 14028s, D23580 and SL1344, but not in pSLT. More work and experiments are needed to further verify the functional significance of these deletions.

SNPs in the pSTUK-100 coding regions were also examined. Two nonsynonymous SNPs are pSTUK-100 specific and located within *trbB* (CCC changed to TCC) and *spvD* (TGC changed to GGC). Most of the SNPs detected in pSTUK-100 were also observed in a subset of the other studied plasmids. For example, we found SNPs within the *tra* regions compared to virulence plasmids from D23580 and SL1344, but not when using virulence plasmids from LT2 or 14028s as the reference. Nonsynonymous SNPs in *traE* (CCG changed to TCG) and *orf6* (CAG changed to CAT) were detected only for pSLT. Other plasmid pSTUK-100 genes, such as fimbrial gene *pefD*, DNA replication gene *repA2*, and *sdiA*-regulated gene *srgB*, were also found to carry SNPs.

In addition, one VNTR locus was detected with copy number of 12.2, 10.2, 7.2, 9.2, and 7.2 in the virulence plasmids from UK-1, LT2, 14028s, D23580, and SL1344, respectively. This VNTR is located 29 bp downstream of a gene encoding a putative inner membrane protein STMUK_p038 and 630 bp upstream of the gene encoding the single-strand binding protein SsbB.

Identification of all possible variations in the virulence-associated plasmid of *S.* Typhimurium should provide us with important clues to the virulence and diversity of the UK-1 strain. It is of interest to note that when the virulence plasmids of strains LT2, SL1344 and 14028s were interchanged, there was no effect on the competitive index in co-immunized BALB/c mice, indicating that, at least for these three strains in this model, the virulence plasmids were equivalent [76]. It will be interesting to perform a similar analysis with the UK-1 virulence plasmid to determine whether pSTUK-100 provides an advantage to other strains and, if so, to examine what role the observed polymorphisms in pSTUK-100 may play in virulence.

### Phylogenetic Analysis

The phylogenetic tree based on the conserved regions of whole genomes represents the evolutionary relationship of the five *S. *Typhimurium strains (Fig. 2A). The tree shows that all divergent events among UK-1, 14028s, D23580 and SL1344 occurred later than the time that virulent strains diverged from avirulent strains, indicating that LT2 shared a most recent common ancestor with the other Typhimurium strains. Interestingly, pairwise comparison of the SNPs among the five genomes show that D23580 harbors the most SNPs among the five strains, which indicates that D23580 is a highly divergent isolate with extensive genetic diversity. There is also evidence of genome degradation in UK-1 (23 pseudogenes detected) when compared with other *S. *Typhimurium genome sequences, except D23580. The amount of genome degradation is greater in the invasive, multidrug-resistant host-adapted strain D23580 (77 pseudogenes) that emerged in Africa in recent years [31]. The observed variation in D23580 is consistent with the notion that genome evolution (including genome degradation) occurs as *Salmonella* strains undergo progressive adaptation to particular hosts, thus providing a unique window into our understanding of the evolution of host adaptation [77].

The estimated divergence time between LT2 and 14028s suggests that the common ancestor of these *S.* Typhimurium strains existed around 9000 years ago [6]. The phylogeny would be a useful framework for investigating the recent evolution of phenotypic traits such as the acquisition of resistance to bacteriocin [78], a class of antibiotics used to treat salmonellosis and acute gastroenteritis caused by *Salmonella*.

### Summary

We have conducted a thorough comparative genome analysis of UK-1 with other *S.* Typhimurium strains by utilizing a vast array of bioinformatic software tools. Sequencing of the *S.* Typhimurium UK-1 genome and comparative analysis provide key information for evaluating the functional characterization of important genetic determinants of *S.* Typhimurium. It demonstrates that even highly similar *S.* Typhimurium strains could be differentiated once the polymorphic genomic regions are identified and analyzed. Studying these variations may lead to the discovery of new virulence determinants that can be used as targets in the development of novel intervention strategies for both the prevention and treatment of infectious diseases.

## Materials and Methods

### Ethics Statement

Animal studies were carried out in strict accordance with the recommendations in the Guide for the Care and Use of Laboratory Animals of the National Institutes of Health. The protocol was approved by the Arizona State University Animal Care and Use Committee (Protocol Number: 11-1168R).

### Strain Description

*Salmonella* Typhimurium UK-1 (χ3761) is a chicken-passaged isolate of strain χ3663, a highly virulent *S*. Typhimurium strain isolated from an infected horse [9]. A day-of-hatch specific pathogen free white leghorn chick was orally inoculated with strain χ3663. *Salmonella* was isolated from the spleen of the chick three days later. One of the spleen isolates was designated strain χ3761.

### Suicide Plasmids and Mutant Strain Construction

For the ΔSTMUK_2657 deletion, two pairs of primers **YQ-1F(aaatttcatcttctacgccttg)**/**YQ-1R(catcccaattctgttgcacttccttattatg)** and **YQ-2F(agtgcaacagaattgggatggtcaatccct)**/**YQ-2R(tgattatgtttgtctacgaag)** were used to amplify approximately 300-bp upstream and downstream fragments of gene STMUK_2657, respectively, from the χ3761 genome. The two fragments were then fused by PCR using primers **YQ-1F** and **YQ-2R**. The terminal A was added at both ends to the resulting PCR product by GoTaq enzyme (Promega), which was inserted into T-cloning suicide vector pYA4278 [79] to generate plasmid pYA5197 carrying a 324 base-pair deletion of the STMUK_2657 gene (from 2766418 base to 2766084 base). A similar strategy was used to construct plasmid pYA5198 (ΔSTMUK_2664) (**YQ-3F:accgcgttcttctggagatg/YQ-3R:caaaagatttcgaaagattttcatttaacg and YQ-4F:aaatctttcgaaatcttttgagaaatggattg/YQ-4R:aagaataagaacccgatcagc**), which carries a 444 bp deletion in the STMUK_2664 gene (from 2771580 base to 2771136 base). The mutations were independently introduced into *S. *Typhimurium χ3761 by allelic exchange by conjugation with *E. coli* strain χ7213 harboring suicide plasmids pYA5197 and pYA5198 to generate ΔSTMUK_2657 (χ11476) and ΔSTMUK_2664 (χ11477), respectively.

### Bacteriophage Typing

Bacteriophage typing was performed by the National Veterinary Services Laboratories in accordance with the method of the Health Protection Agency, London, United Kingdom [80].

### LD_50_ Examination of Virulence of *S.* Typhimurium Strains in Mice

The virulence of the *Salmonella* strains was determined by determining the LD_50_ in mice according to our standard procedure [81]. We examined the virulence of four non-host-adapted strains UK-1, 14028S, and SL1344 by measuring the median lethal doses. Strain D23580 was not available at the time that we performed the experiments. Therefore, D23580 was not included in our analysis. Each strain was grown from a single colony in LB broth overnight at 37°C [82]. One ml of each broth culture was inoculated into 100 ml of pre-warmed fresh LB broth. The cultures were grown in LB with aeration, shaking at 180 rpm to 0.85 OD_600_. Cells were collected by centrifugation at 4100 rpm for 15 minutes at room temperature. Each pellet was resuspended in phosphate buffered saline-gelatin to a dose of approximately 1×10^9^ CFU per 20 µl. The prepared samples were diluted serially to prepare inocula and exact titers determined by plating serial dilutions on LB agar plates. Female BALB/c mice, 6–7 weeks old, were obtained from Charles River Laboratories. Mice were acclimated for 7 days before starting the experiments. 7–8 week old female BALB/c mice were fasted for 4–5 h and inoculated orally with the prepared strain samples. A 20 µl volume containing 10^2^, 10^3^, 10^4^, 10^5^, 10^6^, and 10^7^ CFU of each strain were used to orally inoculate five mice per dose group. Mice were observed daily after inoculation. We repeated this experiment three times.

We used the same strategy to examine the virulence of the ΔSTMUK_2657 and ΔSTMUK_2664 mutants. To evaluate colonization, mice were orally inoculated with 20 µl BSG containing 1×10^9^ CFU each strain. In the first experiment, 20 µl containing 10^3^, 10^4^, 10^5^ or 10^6^ CFU of UK-1 wild-type strain were used to orally inoculate five mice per dose group and 20 µl containing 10^4^, 10^5^ or 10^6^ CFU of each of the two mutant strains was used to inoculate 2 mice per dose group. After the preliminary experiment, three repeats were performed. For each repeat, 20 µl containing 10^2^, 10^3^, 10^4^, 10^5^ or 10^6^ CFU of each strain was used to orally inoculate groups of five mice. We used all data combined in our analyses.

Treatment groups receiving similar doses were combined, using the weighted average of the exact CFU/group as the dose for the combined group. This resulted in more mice per dose group. The LD_50_ and its upper and lower 95% confidence limits were determined using the trimmed Spearman-Karber method [83]. Calculations were done by a DOS program available from the U.S. EPA (http://www.epa.gov/eerd/stat2.htm#tsk) that also trimmed the data when appropriate.

### Genomic Data Source

The complete *S.* Typhimurium UK-1 genome has been deposited into GenBank under accession numbers CP002614 for the bacterial chromosome and CP002615 for the plasmid. As of this writing there were four other *S.* Typhimurium genomes available in the public database. Annotation files of LT2 (Accession no: NC_003197 and NC_003277), 14028s (Accession no: CP001363 and CP001362), and D23580 (Accession no: FN424405 and FN432031) were obtained from NCBI GenBank (ftp://ftp.ncbi.nih.gov/genomes/Bacteria). SL1344 was obtained from the Sanger Institute (ftp://ftp.sanger.ac.uk/pub/pathogens/Salmonella/).

### Pseudogene Identification

Due to the high similarity between the five *S.* Typhimurium genomes, the output from SPALN was further analyzed to identify pseudogenes since SPALN mapped any homologous genes from the reference genome onto the UK-1 genome [84]. We first mapped all gene sequences from LT2, 14028s, D23580 and SL1344 onto the UK-1 genome, respectively. SPALN identified all the orthologous genes (or segments) found in the UK-1 genome. We determined anchors (pairs of genes, one from UK-1, and the other one from the reference genome) by BLASTP using identity greater than 80 to remove lower probability matches [85]. Then we aligned each pair of two anchoring genes using MUSCLE [86]. Possible gene-inactivating mutations, including insertions, deletions, pre-mature stop codons, or remnants of genes in the UK-1 genome were inferred based on the annotated gene in the reference genome. All predicted pseudogenes were manually inspected for consistency regarding gene synteny (both homology and order).

To ensure high accuracy of this method for the identification of pseudogenes, we also compared the pseudogenes in 14028s and D23580 identified by our method, with those detected in the studies of Jarvik et al. and Kingsley et al., respectively [6], [31]. As expected, the two lists of pseudogenes are highly consistent, lending a degree of confidence to our method of identifying pseudogenes in UK-1. All the pseudogenes caused by substitutions or small indels (<6 bp), were verified by Sanger sequencing of PCRs.

### Identification of SNPs

The genomic sequence for SNP analysis did not include phage or repetitive regions in order to minimize the noise in highly active regions. SNPs between any two *S.* Typhimurium strains were detected using the NUCmer and shown-snps programs in the MUMmer 3 package [87]. SNPs and small indels (length of insertion or deletion greater than one nucleotide) located inside both coding and non-coding regions were determined with PERL scripts. The SNPs within coding regions were then classified as synonymous or nonsynonymous by the method described by Nei and Gojobori [88] using a modified SNAP program [89].

### Identification of Variation Number of Tandem Repeats

A tandem repeat is another type of mutational event, which consists of two or more contiguous, approximate copies of a pattern of nucleotides. We determined the tandem repeats in each genomic sequence by employing Tandem Repeats Finder [57], with a strict threshold of minimum alignment score of 80, and other default parameters. Copy number refers to the number of repeat copies aligned with the consensus pattern.

### CRISPR Analysis

CRISPR loci were detected using CRISPRFinder [90]. Non-coding sequences located at the 5′-end of the first identified CRISPR repeat for each locus were selected as putative leader sequence. The Hidden Markov models (HMMs) for the 45 Cas protein families were obtained from TIGRFAM [91]. Identification of *cas* genes was performed using hmmscan in hmmer-3.0 [92] and BLASTP [85]. Similarities to spacers were searched for against the nt database obtained from NCBI (ftp.ncbi.nih.gov/BLAST/db/) using the BLASTN program by turning off the filter setting for short/near exact matches and a word size of 7 [85].

### Phylogeny

*Salmonella* Typhi Ty2 (Accession no: NC_004631) was used to root the *S.* Typhimurium phylogeny. We used whole genomic sequences excluding phage and repetitive regions to infer the phylogenetic relationship of *S.* Typhimurium genomes. Whole genome alignments were performed with the progressive alignment method in MAUVE [93]. Ambiguously aligned regions were omitted using Gblocks ver. 0.91b [94]. The phylogeny was inferred with 4,270,594 nucleotides using two methods: MEGA5 for Neighbor-Joining (NJ) [95] and RAXML for Maximum-Likelihood (ML) [96]. The evolutionary distances were computed using the Tamura-Nei method with Gamma distributed model, and the GTR+GAMMA+I model was used to include an estimate of the proportion of invariable sites [97]. The extremely high degree of similarity among the five *S.* Typhimurium genomes makes phylogenetic inference difficult since appropriate phylogenetic markers are hard to find. To avoid any bias of the selected phylogenetic marker, we also constructed a phylogenetic tree based on the number of SNPs across whole genomes excluding phage and replicated regions. The tree based on SNPs is the same as the topologies obtained by NJ and ML methods on whole genomic sequence, which further proves the phylogenetic relationship we have obtained is the best one for *S.* Typhimurium strains.

## Supporting Information

Figure S1 **Distribution of orthologous ORFs in UK-1, LT2, 14028s, D23580, and SL1344.** Each Venn diagram shows the number of genes unique in UK-1 or shared between LT2 and one of the other three *S.* Typhimurium genomes.
(TIF)

Figure S2 **Alignment of the genome segment of the five** ***S.*** **Typhimurium virulence plasmids.** The region includes the UK-1 unique deletion adjacent to the operon *spvRABCD*. The sequence alignments were generated in MAUVE. The regions conserved among all genomes were colored in purple and the regions conserved among subsets of the genomes used other colors. If the areas contain sequence elements not aligned, those were marked in white. Regions that were not colored indicate no detectable homology among the five genomes in MAUVE. The predicted genes in these regions are shown with red solid arrays. The names of genes are indicated with the strain name (PUK indicates pSTUK-100, PLT indicates PSLT, 14- indicates the plasmid of 14028s, BT indicates the plasmid of D23580, and SLP indicates plasmid 1 of SL1344) followed by its locus number obtained from each of the annotation files. The putative adhesin gene lost in pSTUK-100 is marked in red in the alignment.
(TIF)

Table S1 **Virulence comparison of the UK-1 specific gene mutants with the UK-1 parent in mice.**
(DOC)

Table S2 **Pseudogenes detected in UK-1.**
(DOC)

Table S3 **Table of polymorphisms including synonymous and nonsynonymous SNPs detected in the UK-1 strain by referring to the other four genomes.**
(DOC)

Table S4 **Insertions detected in the UK-1 strain by referring to the other four** ***S.*** **Typhimurium strains.**
(DOC)

Table S5 **43 VNTRs identified in the five** ***S.*** **Typhimurium strains.**
(DOC)
