# Supplementary figures and images for: Comparative Genome Analysis of the High Pathogenicity Salmonella Typhimurium Strain UK-1
Source: PLoS One. 2012 Jul 6;7(7):e40645. doi: 10.1371/journal.pone.0040645 (PMC3391293; doi:10.1371/journal.pone.0040645)

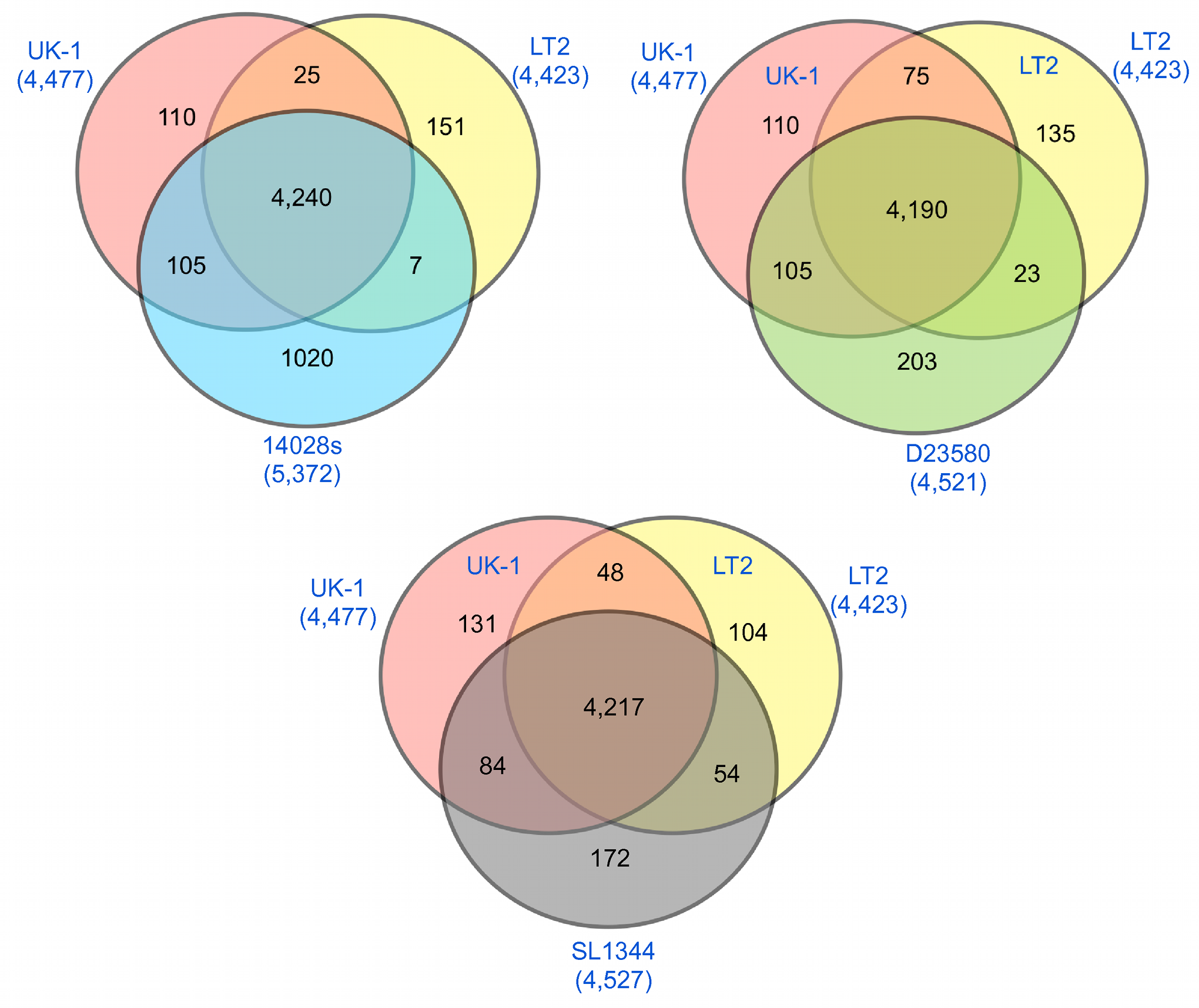

Supplement: Figure S1 — Distribution of orthologous ORFs in UK-1, LT2, 14028s, D23580, and SL1344. Each Venn diagram shows the number of genes unique in UK-1 or shared between LT2 and one of the other three S. Typhimurium genomes. (TIF) [file pone.0040645.s001.tif]

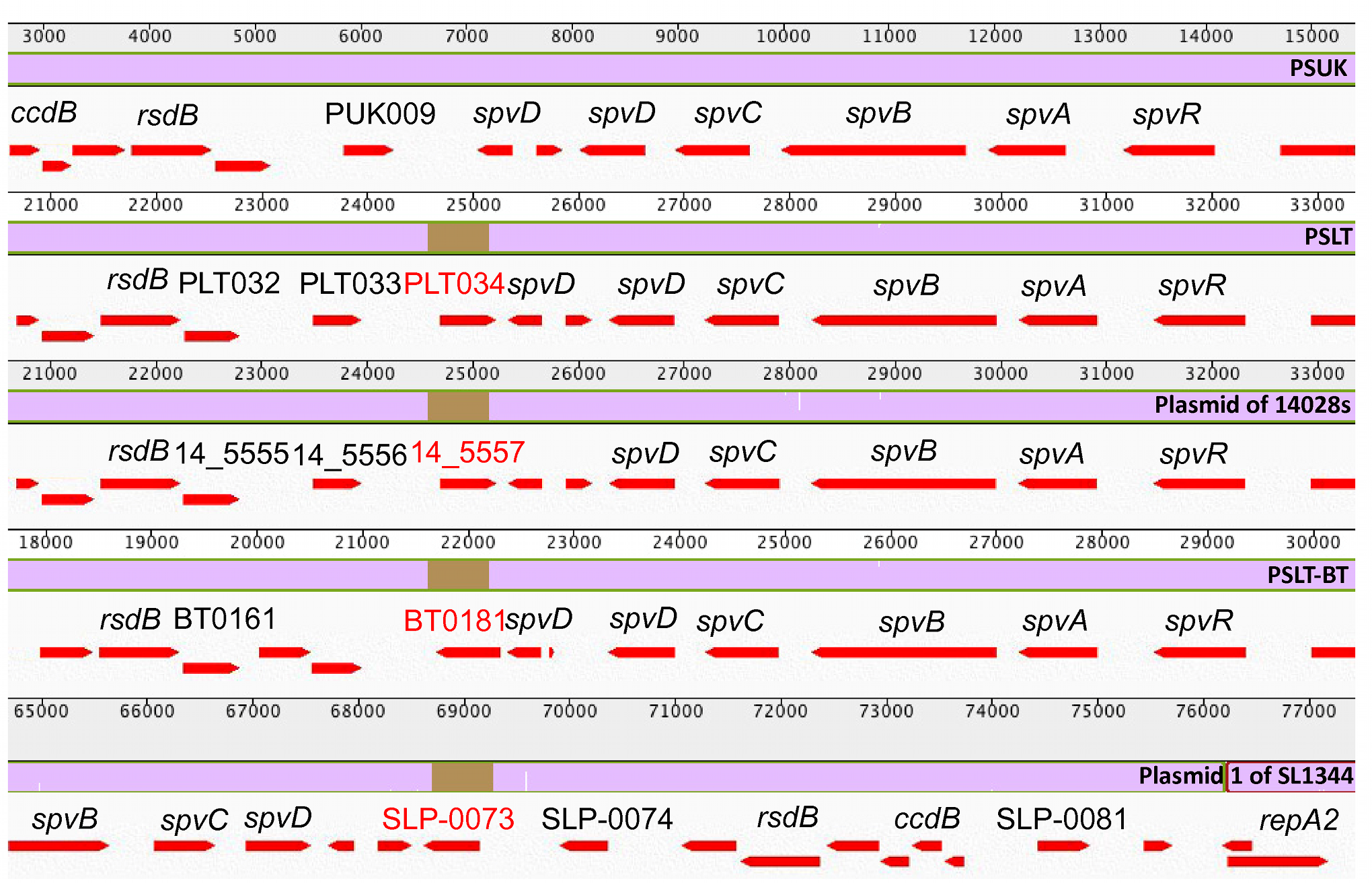

Supplement: Figure S2 — Alignment of the genome segment of the five S. Typhimurium virulence plasmids. The region includes the UK-1 unique deletion adjacent to the operon spvRABCD. The sequence alignments were generated in MAUVE. The regions conserved among all genomes were colored in purple and the regions conserved among subsets of the genomes used other colors. If the areas contain sequence elements not aligned, those were marked in white. Regions that were not colored indicate no detectable homology among the five genomes in MAUVE. The predicted genes in these regions are shown with red solid arrays. The names of genes are indicated with the strain name (PUK indicates pSTUK-100, PLT indicates PSLT, 14- indicates the plasmid of 14028s, BT indicates the plasmid of D23580, and SLP indicates plasmid 1 of SL1344) followed by its locus number obtained from each of the annotation files. The putative adhesin gene lost in pSTUK-100 is marked in red in the alignment. (TIF) [file pone.0040645.s002.tif]
