# Supplementary material for: Comparative Genome Analysis of the High Pathogenicity Salmonella Typhimurium Strain UK-1
Source: PLoS One. 2012 Jul 6;7(7):e40645. doi: 10.1371/journal.pone.0040645 (PMC3391293; doi:10.1371/journal.pone.0040645)
Supplement: Table S1 — Virulence comparison of the UK-1 specific gene mutants with the UK-1 parent in mice. (DOC) [file pone.0040645.s003.doc]

**Table S1. Virulence comparison of the UK-1 mutants and the UK-1 parent strain in mice.**

| **Experiments** | **UK-1 parent** | | | | **ΔSTMUK_2657** | | | | **ΔSTMUK_2664** | | | |
| --- | --- | --- | --- | --- | --- | --- | --- | --- | --- | --- | --- | --- |
|  | **dose**  **(CFU)** | **tested** | **survival** | **killed** | **dose**  **(CFU)** | **tested** | **survival** | **killed** | **dose**  **(CFU)** | **tested** | **survival** | **killed** |
| Small scale | 4.00E+03 | 5 | 5 | 0 | 6.40E+04 | 2 | 2 | 0 | 5.60E+04 | 2 | 0 | 2 |
| Small scale | 4.00E+04 | 5 | 2 | 3 | 6.40E+05 | 2 | 0 | 2 | 5.60E+05 | 2 | 0 | 2 |
| Small scale | 4.00E+05 | 5 | 2 | 3 | 6.40E+06 | 2 | 0 | 2 | 5.60E+06 | 2 | 0 | 2 |
| Small scale | 4.00E+06 | 5 | 0 | 5 |  |  |  |  |  |  |  |  |
| 1st repeat | 2.20E+02 | 5 | 5 | 0 | 3.60E+02 | 5 | 5 | 0 | 2.20E+02 | 5 | 5 | 0 |
| 1st repeat | 2.20E+03 | 5 | 4 | 1 | 3.60E+03 | 5 | 5 | 0 | 2.20E+03 | 5 | 3 | 2 |
| 1st repeat | 2.20E+04 | 5 | 2 | 3 | 3.60E+04 | 5 | 4 | 1 | 2.20E+04 | 5 | 3 | 2 |
| 1st repeat | 2.20E+05 | 5 | 3 | 2 | 3.60E+05 | 5 | 3 | 2 | 2.20E+05 | 5 | 0 | 5 |
| 1st repeat | 2.20E+06 | 5 | 0 | 5 | 3.60E+06 | 5 | 5 | 0 | 2.20E+06 | 5 | 0 | 5 |
| 2nd repeat | 2.50E+02 | 5 | 5 | 0 | 2.60E+02 | 5 | 5 | 0 | 2.40E+02 | 5 | 5 | 0 |
| 2nd repeat | 2.50E+03 | 5 | 0 | 5 | 2.60E+03 | 5 | 4 | 1 | 2.40E+03 | 5 | 4 | 1 |
| 2nd repeat | 2.50E+04 | 5 | 1 | 4 | 2.60E+04 | 5 | 0 | 5 | 2.40E+04 | 5 | 1 | 4 |
| 2nd repeat | 2.50E+05 | 5 | 0 | 5 | 2.60E+05 | 5 | 1 | 4 | 2.40E+05 | 5 | 0 | 5 |
| 2nd repeat | 2.50E+06 | 5 | 0 | 5 | 2.60E+06 | 5 | 0 | 5 | 2.40E+06 | 5 | 0 | 5 |
| 3rd repeat | 2.50E+03 | 5 | 1 | 4 | 3.60E+03 | 5 | 3 | 2 | 3.00E+03 | 5 | 4 | 1 |
| 3rd repeat | 2.50E+04 | 5 | 4 | 1 | 3.60E+04 | 5 | 4 | 1 | 3.00E+04 | 5 | 2 | 3 |
| 3rd repeat | 2.50E+05 | 5 | 1 | 4 | 3.60E+05 | 5 | 0 | 5 | 3.00E+05 | 5 | 0 | 5 |
| 3rd repeat | 2.50E+06 | 5 | 0 | 5 | 3.60E+06 | 5 | 0 | 5 | 3.00E+06 | 5 | 0 | 5 |
