# Supplementary material for: Comparative Genome Analysis of the High Pathogenicity Salmonella Typhimurium Strain UK-1
Source: PLoS One. 2012 Jul 6;7(7):e40645. doi: 10.1371/journal.pone.0040645 (PMC3391293; doi:10.1371/journal.pone.0040645)
Supplement: Table S2 — Pseudogenes detected in UK-1. (DOC) [file pone.0040645.s004.doc]

Table S2. Pseudogenes detected in UK-1.

| **Locus tag in LT2** | **Gene Name** | **Locus tag in UK-1** | **Function** | **Description** |
| --- | --- | --- | --- | --- |
| STM2514 | *ratB* | STMUK_2546 | putative outer membrane protein | Single deletion |
| STM0695 | *ybfE* | STMUK_0700 | LexA regulated protein | Stop codon inside |
| STM1570 | *fdnG* | STMUK_1539 | formate dehydrogenase-N alpha subunit | Stop codon inside; pseudogene in LT2 |
| STM1833 |  | STMUK_1806 | hypothetical protein | Stop codon inside |
| STM2758 |  | STMUK_2746 | putative phosphotransferase system IIC component | Stop codon inside |
| STM3677 | *sgbE* | STMUK_3663 | L-ribulose-5-phosphate 4-epimerase | Stop codon inside |
| STM4037 | *fdoG* | STMUK_4021 | formate dehydrogenase alpha subunit | Stop codon inside; pseudogene in LT2 |
| STM4285 | *fdhF* | STMUK_4270 | formate dehydrogenase | Stop codon inside |
| STM0157 | *yacH* | STMUK_0159 | putative outer membrane protein | Frameshift deletions |
| STM1896 |  | STMUK_1876 | putative cytoplasmic protein | Single deletion |
| STM2629 |  | STMUK_2665 | hypothetical protein | 5’ truncated |
| STM1671 |  | STMUK_1639 | putative regulatory protein | Single deletion |
| STM3637 | *lpfD* | STMUK_3624 | LpfD protein precursor, long polar fimbrial protein | 10 bp deletion |
| STM0657 | *ybeU* | STMUK_0662 | putative cytoplasmic protein | Single deletion |
| STM3260 |  | STMUK_3244 | PTS family galactitol-specific enzyme IIC | 117 left in 1374 bp (5' truncation) |
| STM3255 |  | STMUK_3243 | putative phosphotransferase system fructose-specific component IIB | 792 in 1428 bp (3' truncation) |
| STM3113 | *nupG* | STMUK_3101 | nucleoside transport | 231 of 1257 bp left from the nupG LT2 (3’ truncation) |
| STM0612 |  | STMUK_0617 | putative hydrogenase protein | Large deletion within genes |
| STM2124 | *alkA* | STMUK_2154 | 3-methyl-adenine DNA glycosylase II | Single deletion |
| ^a^ STM2911 |  | STMUK_2900 | putative permease | Single deletion |
|  |  | STMUK_3832 | hypothetical protein; putative phage integrase | Similar to STM14_4639 from 14028s (pseudo), STM_MW38291 from D23580 and SL1344 from SL1344 |
|  |  | STMUK_4439 | hypothetical protein; similar to PTS system, trehalose-specific IIBC component | Similar to STM14_5346 from 14028s (pseudo) and STM_MW43991 from D23580 (pseudo) and SL4385 from SL1344 (pseudo) |
| PSLT002 |  | STMUK_p081 | putative phospholipase D | Single deletion |

^a^ UK-1 specific pseudogene.
