# Supplementary material for: Comparative Genome Analysis of the High Pathogenicity Salmonella Typhimurium Strain UK-1
Source: PLoS One. 2012 Jul 6;7(7):e40645. doi: 10.1371/journal.pone.0040645 (PMC3391293; doi:10.1371/journal.pone.0040645)
Supplement: Table S3 — Table of polymorphisms including synonymous and nonsynonymous SNPs detected in the UK-1 strain by referring to the other four genomes. (DOC) [file pone.0040645.s005.doc]

Table S3. Table of polymorphisms including synonymous and nonsynonymous SNPs detected in the UK-1 strain by referring to other four genomes.

| **COG** | **Gene** | **Group** | **Amino acid change(s)** | **Reference strains** | **Virulence detected by microarray studies ^a^** | | | |
| --- | --- | --- | --- | --- | --- | --- | --- | --- |
|  |  |  |  |  | **Chan et al** | **Chaudhuri et al** | **Lawley et al** | **Santiviago et al** |
| C | atpA | four-strains | L -> I; S -> S; | 14028S; D23580; LT2; SL1344 | 0 | 1 | 0 | 0 |
| C | hycI | four-strains | R -> R; D -> Y; | 14028S; D23580; LT2; SL1344 | 0 | 0 | 0 | 0 |
| C | narZ | four-strains | E -> E; A -> T; | 14028S; D23580; LT2; SL1344 | 0 | 0 | 0 | 0 |
| C | STMUK_2559 | four-strains | K -> E; | 14028S; D23580; LT2; SL1344 | 0 | 0 | 0 | 0 |
| D | ftsX | four-strains | Q -> Q; | 14028S; D23580; LT2; SL1344 | 0 | 0 | 0 | 0 |
| E | gcvT | four-strains | I -> T; | 14028S; D23580; LT2; SL1344 | 0 | 0 | 0 | 0 |
| E | gltK | four-strains | L -> L; | 14028S; D23580; LT2; SL1344 | 1 | 0 | 0 | 0 |
| E | metE | four-strains | A -> A; L -> L; | 14028S; D23580; LT2; SL1344 | 0 | 1 | 0 | 0 |
| E | selD | four-strains | C -> R; S -> S; | 14028S; D23580; LT2; SL1344 | 0 | 0 | 0 | 0 |
| E | STMUK_3116 | four-strains | G -> E; | 14028S; D23580; LT2; SL1344 | 0 | 0 | 1 | 0 |
| E | STMUK_4451 | four-strains | T -> T; | 14028S; D23580; LT2; SL1344 | 0 | 0 | 0 | 0 |
| F | purH | four-strains | S -> G; | 14028S; D23580; LT2; SL1344 | 0 | 1 | 0 | 0 |
| F | pyrH | four-strains | L -> L; | 14028S; D23580; LT2; SL1344 | 0 | 0 | 0 | 0 |
| F | STMUK_1297 | four-strains | S -> F; | 14028S; D23580; LT2; SL1344 | 0 | 0 | 0 | 0 |
| G | fruF | four-strains | P -> P; | 14028S; D23580; LT2; SL1344 | 0 | 0 | 0 | 0 |
| G | fucA | four-strains | V -> A; | 14028S; D23580; LT2; SL1344 | 0 | 0 | 0 | 0 |
| G | hpaX | four-strains | A -> A; H -> H; S -> Y; | 14028S; D23580; LT2; SL1344 | 0 | 0 | 0 | 0 |
| G | malZ | four-strains | P -> P; | 14028S; D23580; LT2; SL1344 | 0 | 0 | 0 | 0 |
| G | rbsD | four-strains | V -> A; | 14028S; D23580; LT2; SL1344 | 0 | 0 | 0 | 0 |
| G | rhaD | four-strains | S -> T; | 14028S; D23580; LT2; SL1344 | 0 | 0 | 0 | 0 |
| G | sgbE | four-strains | Z -> W; | 14028S; D23580; LT2; SL1344 | 0 | 0 | 0 | 0 |
| G | STMUK_2746 | four-strains | L -> F; Z -> Q; | 14028S; D23580; LT2; SL1344 | 0 | 0 | 0 | 0 |
| G | treA | four-strains | G -> G; Y -> N; | 14028S; D23580; LT2; SL1344 | 0 | 0 | 0 | 0 |
| G | ugpA | four-strains | I -> L; | 14028S; D23580; LT2; SL1344 | 0 | 0 | 0 | 0 |
| G | ygbM | four-strains | A -> A; | 14028S; D23580; LT2; SL1344 | 0 | 0 | 0 | 0 |
| I | apeE | four-strains | C -> C; I -> L; | 14028S; D23580; LT2; SL1344 | 0 | 0 | 0 | 0 |
| I | caiC | four-strains | R -> S; | 14028S; D23580; LT2; SL1344 | 0 | 0 | 0 | 0 |
| I | fadJ | four-strains | E -> A; | 14028S; D23580; LT2; SL1344 | 0 | 0 | 0 | 0 |
| K | araC | four-strains | L -> L; | 14028S; D23580; LT2; SL1344 | 0 | 0 | 0 | 0 |
| K | leuO | four-strains | H -> R; | 14028S; D23580; LT2; SL1344 | 0 | 0 | 1 | 0 |
| K | lrp | four-strains | G -> G; | 14028S; D23580; LT2; SL1344 | 0 | 0 | 0 | 0 |
| K | STMUK_4409 | four-strains | I -> I; | 14028S; D23580; LT2; SL1344 | 0 | 0 | 0 | 0 |
| L | polB | four-strains | L -> L; Q -> Q; Y -> Y; | 14028S; D23580; LT2; SL1344 | 0 | 0 | 0 | 0 |
| L | yfaO | four-strains | H -> N; | 14028S; D23580; LT2; SL1344 | 0 | 0 | 0 | 0 |
| M | ftsQ | four-strains | M -> V; I -> I; | 14028S; D23580; LT2; SL1344 | 0 | 0 | 0 | 0 |
| M | ompN | four-strains | T -> A; | 14028S; D23580; LT2; SL1344 | 0 | 0 | 0 | 0 |
| M | tonB | four-strains | T -> T; | 14028S; D23580; LT2; SL1344 | 0 | 0 | 0 | 0 |
| N | aer | four-strains | V -> I; | 14028S; D23580; LT2; SL1344 | 0 | 0 | 0 | 0 |
| N | flgD | four-strains | D -> G; | 14028S; D23580; LT2; SL1344 | 0 | 0 | 0 | 0 |
| N | flgL | four-strains | N -> S; | 14028S; D23580; LT2; SL1344 | 0 | 0 | 0 | 0 |
| N | stdB | four-strains | L -> P; G -> R; G -> W; | 14028S; D23580; LT2; SL1344 | 0 | 0 | 0 | 1 |
| N | yidC | four-strains | M -> L; | 14028S; D23580; LT2; SL1344 | 0 | 0 | 0 | 0 |
| P | iroN | four-strains | L -> M; A -> V; | 14028S; D23580; LT2; SL1344 | 0 | 0 | 0 | 1 |
| P | narU | four-strains | H -> Q; | 14028S; D23580; LT2; SL1344 | 1 | 0 | 0 | 0 |
| P | thiI | four-strains | L -> P; N -> S; | 14028S; D23580; LT2; SL1344 | 0 | 0 | 0 | 0 |
| Q | acrE | four-strains | P -> S; | 14028S; D23580; LT2; SL1344 | 0 | 0 | 0 | 0 |
| R | bioH | four-strains | A -> A; | 14028S; D23580; LT2; SL1344 | 0 | 0 | 0 | 0 |
| R | STMUK_2564 | four-strains | V -> A; P -> S; | 14028S; D23580; LT2; SL1344 | 0 | 0 | 0 | 0 |
| S | pduO | four-strains | T -> A; | 14028S; D23580; LT2; SL1344 | 0 | 0 | 0 | 0 |
| S | pipB2 | four-strains | I -> S; | 14028S; D23580; LT2; SL1344 | 0 | 0 | 0 | 0 |
| S | STMUK_0270 | four-strains | I -> V; L -> F; | 14028S; D23580; LT2; SL1344 | 0 | 0 | 0 | 0 |
| S | STMUK_1291 | four-strains | L -> L; | 14028S; D23580; LT2; SL1344 | 0 | 0 | 0 | 0 |
| S | STMUK_2793 | four-strains | G -> S; | 14028S; D23580; LT2; SL1344 | 0 | 0 | 0 | 0 |
| S | ycbB | four-strains | L -> L; P -> L; | 14028S; D23580; LT2; SL1344 | 0 | 0 | 0 | 0 |
| T | rcsC | four-strains | E -> E; | 14028S; D23580; LT2; SL1344 | 0 | 0 | 0 | 0 |
| T | STMUK_2535 | four-strains | S -> A; V -> V; | 14028S; D23580; LT2; SL1344 | 0 | 0 | 0 | 0 |
| T | yjcC | four-strains | K -> N; | 14028S; D23580; LT2; SL1344 | 0 | 0 | 0 | 0 |
| - | nadR | four-strains | F -> F; | 14028S; D23580; LT2; SL1344 | 0 | 0 | 0 | 0 |
| - | pduU | four-strains | V -> A; | 14028S; D23580; LT2; SL1344 | 0 | 0 | 0 | 0 |
| - | safA | four-strains | S -> P; F -> L; | 14028S; D23580; LT2; SL1344 | 0 | 0 | 0 | 0 |
| - | STMUK_0279 | four-strains | G -> G; | 14028S; D23580; LT2; SL1344 | 0 | 0 | 0 | 0 |
| - | yggN | four-strains | S -> R; | 14028S; D23580; LT2; SL1344 | 0 | 1 | 0 | 0 |
| C | citD | three-strains | Q -> L; | D23580; LT2; SL1344 | 0 | 0 | 0 | 0 |
| C | napA | three-strains | D -> E; | D23580; LT2; SL1344 | 0 | 0 | 0 | 0 |
| C | STMUK_0367 | three-strains | C -> R; | D23580; LT2; SL1344 | 0 | 0 | 0 | 0 |
| C | STMUK_0767 | three-strains | L -> V; | D23580; LT2; SL1344 | 0 | 0 | 0 | 0 |
| C | STMUK_2829 | three-strains | E -> E; H -> Q; G -> G; | D23580; LT2; SL1344 | 0 | 0 | 0 | 0 |
| C | yhdH | three-strains | L -> L; | D23580; LT2; SL1344 | 0 | 0 | 0 | 0 |
| D | ydaO | three-strains | K -> E; | D23580; LT2; SL1344 | 0 | 0 | 0 | 0 |
| E | dapD | three-strains | P -> A; | D23580; LT2; SL1344 | 0 | 0 | 0 | 0 |
| E | ilvI | three-strains | A -> A; | D23580; LT2; SL1344 | 0 | 0 | 0 | 0 |
| E | ldcC | three-strains | A -> A; | D23580; LT2; SL1344 | 0 | 0 | 0 | 0 |
| E | tdcG | three-strains | D -> D; M -> I; | D23580; LT2; SL1344 | 0 | 0 | 0 | 0 |
| G | cpsG | three-strains | I -> T; | D23580; LT2; SL1344 | 0 | 0 | 0 | 0 |
| G | mdfA | three-strains | T -> A; P -> T; V -> M; | D23580; LT2; SL1344 | 1 | 0 | 1 | 0 |
| G | mglB | three-strains | N -> N; | D23580; LT2; SL1344 | 0 | 0 | 0 | 0 |
| G | pfkA | three-strains | E -> E; | D23580; LT2; SL1344 | 0 | 0 | 0 | 0 |
| G | STMUK_3124 | three-strains | A -> A; | D23580; LT2; SL1344 | 0 | 0 | 0 | 0 |
| G | STMUK_3757 | three-strains | H -> R; | D23580; LT2; SL1344 | 0 | 0 | 0 | 0 |
| G | tktB | three-strains | G -> G; R -> S; A -> A; A -> V; | D23580; LT2; SL1344 | 0 | 0 | 0 | 0 |
| G | ulaD | three-strains | T -> A; | D23580; LT2; SL1344 | 0 | 0 | 0 | 0 |
| H | thiC | three-strains | N -> Y; | D23580; LT2; SL1344 | 0 | 0 | 0 | 0 |
| H | yfaW | three-strains | T -> K; | D23580; LT2; SL1344 | 0 | 0 | 0 | 0 |
| J | lysS | three-strains | T -> T; | D23580; LT2; SL1344 | 0 | 0 | 0 | 0 |
| K | nhaR | three-strains | F -> L; | D23580; LT2; SL1344 | 0 | 0 | 0 | 0 |
| K | nusA | three-strains | G -> G; L -> L; | D23580; LT2; SL1344 | 0 | 0 | 0 | 0 |
| K | STMUK_1588 | three-strains | V -> A; | D23580; LT2; SL1344 | 0 | 0 | 0 | 0 |
| K | STMUK_4299 | three-strains | L -> L; L -> P; | D23580; LT2; SL1344 | 1 | 0 | 0 | 1 |
| K | ydhM | three-strains | C -> S; | D23580; LT2; SL1344 | 0 | 0 | 0 | 0 |
| L | sbcC | three-strains | Q -> Q; | D23580; LT2; SL1344 | 0 | 0 | 0 | 0 |
| L | STMUK_4481 | three-strains | L -> L; R -> R; P -> L; | D23580; LT2; SL1344 | 1 | 1 | 0 | 0 |
| L | yjjV | three-strains | Y -> Y; | D23580; LT2; SL1344 | 0 | 0 | 0 | 0 |
| M | mtgA | three-strains | L -> L; | D23580; LT2; SL1344 | 0 | 0 | 0 | 0 |
| M | shdA | three-strains | Z -> R; I -> I; W -> R; L -> L; | D23580; LT2; SL1344 | 0 | 0 | 0 | 0 |
| M | STMUK_4202 | three-strains | D -> G; Q -> R; | D23580; LT2; SL1344 | 0 | 0 | 0 | 0 |
| M | STMUK_4526 | three-strains | G -> E; | D23580; LT2; SL1344 | 0 | 0 | 0 | 0 |
| N | cheA | three-strains | S -> S; R -> R; | D23580; LT2; SL1344 | 0 | 0 | 0 | 0 |
| N | stiB | three-strains | N -> S; | 14028S; D23580; LT2 | 0 | 0 | 0 | 0 |
| P | ftn | three-strains | T -> A; | D23580; LT2; SL1344 | 1 | 0 | 0 | 0 |
| P | STMUK_3061 | three-strains | V -> F; | D23580; LT2; SL1344 | 0 | 0 | 0 | 0 |
| Q | acrD | three-strains | P -> T; L -> L; | D23580; LT2; SL1344 | 0 | 0 | 0 | 0 |
| Q | acrF | three-strains | N -> D; S -> N; | D23580; LT2; SL1344 | 0 | 0 | 0 | 0 |
| Q | cydD | three-strains | S -> P; | D23580; LT2; SL1344 | 0 | 0 | 0 | 0 |
| Q | hpaG | three-strains | R -> R; | D23580; LT2; SL1344 | 1 | 0 | 0 | 0 |
| Q | mdlB | three-strains | L -> P; G -> G; | D23580; LT2; SL1344 | 0 | 0 | 0 | 0 |
| R | bscC | three-strains | L -> L; R -> L; | D23580; LT2; SL1344 | 1 | 0 | 0 | 0 |
| S | erfK | three-strains | V -> A; | D23580; LT2; SL1344 | 0 | 0 | 0 | 0 |
| S | ygbK | three-strains | S -> G; R -> R; | D23580; LT2; SL1344 | 0 | 0 | 0 | 0 |
| S | ygiF | three-strains | R -> R; A -> A; | D23580; LT2; SL1344 | 0 | 0 | 0 | 0 |
| S | yhdP | three-strains | L -> F; | D23580; LT2; SL1344 | 0 | 1 | 0 | 0 |
| S | yhfK | three-strains | A -> A; P -> P; D -> A; | 14028S; D23580; LT2 | 0 | 1 | 0 | 0 |
| - | chaB | three-strains | T -> A; | D23580; LT2; SL1344 | 0 | 0 | 0 | 0 |
| - | kduI | three-strains | I -> V; A -> A; | D23580; LT2; SL1344 | 0 | 0 | 0 | 0 |
| - | pduT | three-strains | S -> G; | D23580; LT2; SL1344 | 0 | 0 | 0 | 0 |
| - | sgaT | three-strains | G -> G; R -> G; S -> S; | D23580; LT2; SL1344 | 1 | 0 | 0 | 0 |
| - | STMUK_0017 | three-strains | K -> E; | D23580; LT2; SL1344 | 0 | 0 | 0 | 0 |
| - | STMUK_2742 | three-strains | I -> S; G -> E; | D23580; LT2; SL1344 | 0 | 0 | 0 | 0 |
| - | STMUK_4246 | three-strains | V -> V; L -> L; T -> S; P -> S; E -> E; R -> R; | 14028S; D23580; LT2 | 1 | 1 | 0 | 1 |
| - | yacH | three-strains | C -> R; V -> V; | D23580; LT2; SL1344 | 0 | 0 | 0 | 0 |
| - | yaiA | three-strains | T -> I; | D23580; LT2; SL1344 | 0 | 0 | 0 | 0 |
| - | ydgA | three-strains | R -> Q; | D23580; LT2; SL1344 | 0 | 0 | 0 | 0 |
| - | yhiH | three-strains | N -> N; H -> Y; | D23580; LT2; SL1344 | 0 | 0 | 0 | 0 |
| - | yicH | three-strains | L -> L; | D23580; LT2; SL1344 | 0 | 0 | 0 | 0 |
| - | yqjE | three-strains | E -> D; | D23580; LT2; SL1344 | 0 | 0 | 0 | 0 |
| - | fdhF | two-strains | V -> A; G -> S; | 14028S; LT2 | 0 | 0 | 0 | 0 |
| - | fixC | two-strains | S -> L; L -> L; | LT2; SL1344 | 0 | 0 | 0 | 0 |
| C | glpK | two-strains | L -> L; T -> I; | D23580; LT2 | 0 | 0 | 0 | 0 |
| C | hmpA | two-strains | R -> C; V -> G; A -> S; | D23580; LT2 | 0 | 0 | 0 | 0 |
| C | ndh | two-strains | M -> V; N -> K; | D23580; LT2 | 0 | 0 | 0 | 0 |
| C | nuoL | two-strains | A -> V; F -> F; A -> A; | 14028S; LT2 | 0 | 1 | 0 | 0 |
| C | ugpQ | two-strains | Q -> L; A -> A; | D23580; LT2 | 0 | 0 | 0 | 0 |
| C | ydiQ | two-strains | L -> R; V -> A; A -> V; | LT2; SL1344 | 1 | 0 | 0 | 0 |
| C | aegA | two-strains | R -> H; N -> N; | D23580; LT2 | 0 | 0 | 0 | 0 |
| C | argD | two-strains | F -> L; T -> M; | 14028S; SL1344 | 0 | 1 | 0 | 0 |
| E | nifS | two-strains | L -> L; E -> E; I -> V; | D23580; SL1344 | 0 | 0 | 0 | 0 |
| E | poxB | two-strains | V -> A; R -> H; L -> L; | D23580; LT2 | 0 | 0 | 0 | 0 |
| E | sdaB | two-strains | S -> R; T -> T; A -> A; L -> V; | D23580; LT2 | 0 | 0 | 0 | 0 |
| E | speC | two-strains | G -> E; A -> V; | D23580; SL1344 | 0 | 0 | 0 | 0 |
| E | STMUK_1096 | two-strains | T -> T; V -> L; | 14028S; D23580 | 0 | 0 | 0 | 0 |
| E | STMUK_1222 | two-strains | K -> R; G -> G; | D23580; SL1344 | 0 | 0 | 0 | 0 |
| E | yabN | two-strains | E -> K; L -> L; | LT2; SL1344 | 0 | 0 | 0 | 0 |
| E | nrdE | two-strains | G -> E; Q -> H; | D23580; SL1344 | 1 | 0 | 0 | 0 |
| E | gntU | two-strains | L -> L; R -> C; | D23580; SL1344 | 0 | 0 | 0 | 0 |
| E | STMUK_0151 | two-strains | V -> I; L -> L; | 14028S; D23580 | 1 | 0 | 0 | 0 |
| F | STMUK_3122 | two-strains | S -> S; G -> G; | D23580; SL1344 | 0 | 0 | 0 | 0 |
| G | uhpC | two-strains | G -> G; V -> M; | D23580; SL1344 | 0 | 0 | 0 | 0 |
| G | ydeV | two-strains | H -> Y; A -> T; | LT2; SL1344 | 0 | 0 | 0 | 0 |
| G | ydiM | two-strains | G -> S; S -> L; | D23580; SL1344 | 0 | 0 | 0 | 0 |
| G | cbiD | two-strains | G -> G; G -> A; | D23580; SL1344 | 0 | 0 | 0 | 0 |
| G | hemE | two-strains | H -> Y; Q -> R; | D23580; LT2 | 0 | 0 | 0 | 0 |
| G | menC | two-strains | P -> P; L -> L; | D23580; SL1344 | 0 | 0 | 0 | 0 |
| H | pdxA | two-strains | Q -> R; I -> S; | D23580; LT2 | 0 | 0 | 0 | 0 |
| H | ligT | two-strains | A -> S; | D23580; SL1344 | 0 | 0 | 0 | 0 |
| H | rumB | two-strains | Y -> H; D -> D; | LT2; SL1344 | 0 | 0 | 0 | 0 |
| H | tsf | two-strains | A -> T; I -> I; | D23580; LT2 | 0 | 0 | 0 | 0 |
| H | rpoB | two-strains | G -> G; L -> L; R -> R; | D23580; LT2 | 0 | 0 | 0 | 0 |
| J | STMUK_0014 | two-strains | S -> G; S -> S; M -> I; | D23580; LT2 | 0 | 0 | 0 | 0 |
| J | STMUK_2391 | two-strains | G -> D; A -> V; | D23580; SL1344 | 1 | 0 | 0 | 0 |
| J | xylR | two-strains | V -> A; D -> N; P -> Q; | LT2; SL1344 | 0 | 0 | 0 | 0 |
| K | yeeY | two-strains | R -> R; | LT2; SL1344 | 0 | 0 | 0 | 0 |
| K | dnaE | two-strains | V -> V; R -> H; | D23580; SL1344 | 0 | 0 | 0 | 0 |
| K | murE | two-strains | A -> V; T -> T; | 14028S; D23580 | 0 | 0 | 0 | 0 |
| K | rfaF | two-strains | T -> A; Q -> Q; P -> P; G -> G; | LT2; SL1344 | 0 | 1 | 0 | 0 |
| K | STMUK_4087 | two-strains | L -> L; W -> Z; | LT2; SL1344 | 0 | 0 | 0 | 0 |
| L | yjeP | two-strains | A -> T; L -> L; | D23580; LT2 | 1 | 0 | 0 | 0 |
| M | corA | two-strains | L -> L; A -> A; | D23580; SL1344 | 0 | 0 | 0 | 0 |
| M | fepA | two-strains | L -> L; G -> G; A -> T; | D23580; LT2 | 0 | 0 | 0 | 0 |
| M | mgtB | two-strains | V -> A; G -> G; | D23580; LT2 | 1 | 0 | 0 | 1 |
| M | modA | two-strains | A -> A; V -> A; | D23580; LT2 | 0 | 0 | 0 | 0 |
| P | ybiR | two-strains | A -> E; A -> D; | D23580; SL1344 | 0 | 0 | 0 | 0 |
| P | yiiP | two-strains | A -> V; T -> S; | 14028S; D23580 | 0 | 0 | 0 | 0 |
| P | hpaB | two-strains | L -> L; F -> L; | 14028S; SL1344 | 0 | 0 | 0 | 0 |
| P | iroC | two-strains | E -> D; T -> T; | 14028S; SL1344 | 0 | 0 | 0 | 0 |
| P | STMUK_2727 | two-strains | A -> A; V -> V; | 14028S; D23580 | 0 | 0 | 0 | 0 |
| P | STMUK_2560 | two-strains | A -> T; Q -> H; | D23580; LT2 | 0 | 0 | 0 | 0 |
| Q | ydgC | two-strains | V -> A; S -> S; | LT2; SL1344 | 0 | 0 | 0 | 0 |
| Q | STMUK_0288 | two-strains | F -> L; T -> M; | 14028S; LT2 | 0 | 0 | 0 | 0 |
| Q | yjeF | two-strains | V -> V; G -> G; | 14028S; LT2 | 0 | 0 | 0 | 0 |
| R | baeS | two-strains | F -> L; V -> V; | D23580; LT2 | 0 | 0 | 0 | 0 |
| R | cstA | two-strains | G -> G; I -> L; | LT2; SL1344 | 0 | 1 | 0 | 0 |
| S | glnG | two-strains | S -> S; E -> K; | D23580; SL1344 | 0 | 0 | 0 | 0 |
| S | STMUK_3759 | two-strains | E -> E; H -> R; | D23580; LT2 | 0 | 0 | 0 | 0 |
| T | STMUK_4538 | two-strains | A -> A; T -> T; A -> T; | D23580; LT2 | 0 | 0 | 0 | 0 |
| T | yegE | two-strains | G -> R; L -> L; | 14028S; LT2 | 0 | 1 | 0 | 1 |
| T | pduK | two-strains | R -> W; V -> A; | LT2; SL1344 | 0 | 0 | 0 | 0 |
| T | ratA | two-strains | L -> L; F -> L; | D23580; LT2 | 0 | 0 | 0 | 0 |
| T | STMUK_0033 | two-strains | D -> N; L -> L; | D23580; LT2 | 0 | 0 | 0 | 0 |
| T | STMUK_2164 | two-strains | M -> I; A -> S; | D23580; SL1344 | 0 | 0 | 0 | 0 |
| - | STMUK_3154 | two-strains | T -> A; W -> C; | D23580; SL1344 | 0 | 0 | 0 | 0 |
| - | STMUK_3835 | two-strains | F -> F; T -> T; | D23580; SL1344 | 0 | 0 | 0 | 0 |
| - | STMUK_3913 | two-strains | R -> I; T -> A; | D23580; SL1344 | 0 | 1 | 0 | 0 |
| - | aceE | one-strain | P -> P; | LT2 | 1 | 0 | 0 | 0 |
| - | aceF | one-strain | A -> R; | LT2 | 0 | 0 | 0 | 0 |
| - | atpF | one-strain | L -> L; | SL1344 | 1 | 1 | 0 | 0 |
| - | citF | one-strain | S -> R; | SL1344 | 0 | 0 | 0 | 0 |
| C | citF2 | one-strain | A -> V; | D23580 | 0 | 0 | 0 | 0 |
| C | cybB | one-strain | M -> R; | D23580 | 1 | 0 | 0 | 0 |
| C | cyoB | one-strain | L -> L; | D23580 | 0 | 0 | 1 | 0 |
| C | eutD | one-strain | R -> S; | D23580 | 0 | 0 | 0 | 0 |
| C | fdoG | one-strain | G -> G; | D23580 | 0 | 0 | 0 | 0 |
| C | fpr | one-strain | Q -> H; | SL1344 | 0 | 0 | 0 | 0 |
| C | frdD | one-strain | S -> T; | LT2 | 0 | 1 | 0 | 0 |
| C | gabD | one-strain | S -> S; | LT2 | 0 | 0 | 0 | 0 |
| C | glpA | one-strain | R -> Q; | SL1344 | 0 | 0 | 0 | 0 |
| C | gltA | one-strain | A -> T; | LT2 | 0 | 0 | 0 | 0 |
| C | hcp | one-strain | Q -> Q; | SL1344 | 0 | 1 | 0 | 0 |
| C | hybA | one-strain | F -> L; | D23580 | 0 | 0 | 0 | 0 |
| C | hycC | one-strain | G -> A; | LT2 | 0 | 0 | 0 | 0 |
| C | maeB | one-strain | V -> F; | D23580 | 0 | 0 | 0 | 0 |
| C | napG | one-strain | G -> G; | LT2 | 0 | 0 | 0 | 0 |
| C | napH | one-strain | K -> N; | D23580 | 0 | 0 | 0 | 0 |
| C | nfnB | one-strain | E -> E; | SL1344 | 0 | 0 | 1 | 0 |
| C | nuoF | one-strain | R -> R; | 14028S | 0 | 1 | 0 | 0 |
| C | nuoH | one-strain | E -> E; | LT2 | 0 | 1 | 0 | 0 |
| C | pflB | one-strain | L -> L; | LT2 | 0 | 0 | 0 | 0 |
| C | pflD | one-strain | L -> L; | SL1344 | 0 | 0 | 0 | 0 |
| C | sdhA | one-strain | Q -> H; | D23580 | 0 | 1 | 0 | 0 |
| C | sfcA | one-strain | Q -> Q; | D23580 | 0 | 0 | 0 | 0 |
| C | STMUK_0366 | one-strain | M -> I; | D23580 | 0 | 0 | 0 | 0 |
| C | STMUK_0616 | one-strain | V -> V; | D23580 | 0 | 1 | 0 | 0 |
| C | STMUK_0860 | one-strain | D -> N; | D23580 | 1 | 0 | 0 | 0 |
| C | STMUK_0864 | one-strain | G -> G; | 14028S | 1 | 0 | 0 | 0 |
| C | STMUK_1228 | one-strain | E -> Q; | SL1344 | 0 | 0 | 0 | 0 |
| C | STMUK_1423 | one-strain | P -> P; | SL1344 | 0 | 0 | 0 | 0 |
| C | STMUK_1461 | one-strain | G -> D; | D23580 | 0 | 0 | 0 | 0 |
| C | STMUK_1462 | one-strain | R -> H; | SL1344 | 0 | 0 | 0 | 0 |
| C | STMUK_1503 | one-strain | G -> R; | SL1344 | 0 | 0 | 0 | 0 |
| C | STMUK_1525 | one-strain | G -> R; | SL1344 | 0 | 0 | 0 | 0 |
| C | STMUK_1758 | one-strain | G -> G; | LT2 | 1 | 1 | 0 | 0 |
| C | STMUK_1764 | one-strain | Q -> H; | LT2 | 0 | 0 | 0 | 0 |
| C | STMUK_2562 | one-strain | L -> Q; | D23580 | 0 | 0 | 0 | 0 |
| C | STMUK_3069 | one-strain | Q -> R; | LT2 | 0 | 0 | 0 | 0 |
| C | STMUK_4408 | one-strain | G -> S; | LT2 | 0 | 0 | 0 | 0 |
| C | sucC | one-strain | P -> L; | SL1344 | 0 | 0 | 0 | 0 |
| C | ttrA | one-strain | F -> L; A -> A; | D23580 | 0 | 0 | 0 | 0 |
| C | ydiR | one-strain | N -> K; | D23580 | 0 | 0 | 0 | 0 |
| C | ydiS | one-strain | V -> L; | 14028S | 1 | 0 | 0 | 0 |
| C | ydiT | one-strain | A -> T; | D23580 | 0 | 0 | 0 | 0 |
| C | yhbW | one-strain | P -> S; | LT2 | 0 | 0 | 0 | 0 |
| C | maf | one-strain | I -> I; | D23580 | 0 | 0 | 0 | 0 |
| C | mukB | one-strain | A -> V; T -> T; | D23580 | 0 | 0 | 0 | 0 |
| C | zipA | one-strain | D -> V; | LT2 | 0 | 0 | 0 | 0 |
| C | adi | one-strain | A -> S; | LT2 | 0 | 0 | 0 | 0 |
| D | argT | one-strain | L -> I; | D23580 | 0 | 0 | 0 | 0 |
| D | aroF | one-strain | L -> L; | D23580 | 0 | 0 | 0 | 0 |
| D | aroP | one-strain | A -> T; | D23580 | 0 | 0 | 0 | 0 |
| D | artI | one-strain | H -> Y; | SL1344 | 0 | 0 | 0 | 0 |
| E | cadA | one-strain | F -> F; | D23580 | 0 | 0 | 0 | 0 |
| E | carB | one-strain | G -> G; | SL1344 | 1 | 1 | 0 | 0 |
| E | dadA | one-strain | G -> G; | SL1344 | 0 | 0 | 0 | 0 |
| E | dsdA | one-strain | G -> D; | D23580 | 0 | 0 | 0 | 0 |
| E | gcvP | one-strain | E -> E; | D23580 | 0 | 0 | 0 | 0 |
| E | gdhA | one-strain | A -> A; | D23580 | 0 | 0 | 0 | 0 |
| E | glnH | one-strain | D -> E; | D23580 | 0 | 0 | 0 | 0 |
| E | gltI | one-strain | A -> D; | LT2 | 0 | 0 | 0 | 0 |
| E | gsp | one-strain | D -> E; | 14028S | 0 | 0 | 0 | 0 |
| E | hisG | one-strain | L -> P; | SL1344 | 0 | 0 | 0 | 0 |
| E | hisM | one-strain | A -> T; | D23580 | 0 | 0 | 0 | 0 |
| E | hutH | one-strain | L -> L; | LT2 | 0 | 0 | 0 | 0 |
| E | idnD | one-strain | R -> S; R -> Q; | D23580 | 0 | 0 | 0 | 0 |
| E | ilvD | one-strain | G -> G; | SL1344 | 0 | 0 | 0 | 0 |
| E | livK | one-strain | S -> G; | SL1344 | 0 | 0 | 0 | 0 |
| E | lysC | one-strain | K -> K; | 14028S | 0 | 0 | 0 | 0 |
| E | lysP | one-strain | A -> E; | SL1344 | 0 | 0 | 0 | 0 |
| E | nanA | one-strain | A -> V; | SL1344 | 0 | 0 | 0 | 1 |
| E | oat | one-strain | A -> T; | SL1344 | 0 | 0 | 0 | 0 |
| E | pdxB | one-strain | A -> E; | 14028S | 0 | 0 | 0 | 0 |
| E | pepA | one-strain | G -> D; | SL1344 | 0 | 0 | 0 | 0 |
| E | pepD | one-strain | R -> C; | D23580 | 0 | 0 | 0 | 0 |
| E | pepE | one-strain | A -> A; | SL1344 | 0 | 0 | 0 | 0 |
| E | potH | one-strain | L -> L; | SL1344 | 0 | 0 | 0 | 0 |
| E | prlC | one-strain | A -> T; | D23580 | 0 | 0 | 0 | 0 |
| E | proB | one-strain | R -> R; | D23580 | 0 | 1 | 0 | 0 |
| E | proC | one-strain | A -> T; | 14028S | 0 | 0 | 0 | 0 |
| E | proW | one-strain | G -> E; | SL1344 | 0 | 0 | 0 | 0 |
| E | putP | one-strain | A -> A; | LT2 | 0 | 0 | 0 | 0 |
| E | sdaA | one-strain | H -> R; | LT2 | 0 | 0 | 0 | 0 |
| E | selA | one-strain | L -> F; | SL1344 | 0 | 0 | 0 | 0 |
| E | speA | one-strain | M -> T; | LT2 | 0 | 0 | 0 | 0 |
| E | speD | one-strain | D -> G; | D23580 | 0 | 0 | 0 | 0 |
| E | speF | one-strain | R -> H; | 14028S | 0 | 0 | 0 | 0 |
| E | STMUK_0335 | one-strain | W -> Z; | SL1344 | 0 | 0 | 0 | 0 |
| E | STMUK_0968 | one-strain | G -> G; | LT2 | 0 | 0 | 1 | 0 |
| E | STMUK_1447 | one-strain | T -> A; | D23580 | 0 | 0 | 0 | 0 |
| E | STMUK_1454 | one-strain | D -> A; | D23580 | 0 | 0 | 0 | 0 |
| E | STMUK_1526 | one-strain | S -> S; | D23580 | 0 | 0 | 0 | 0 |
| E | STMUK_2387 | one-strain | V -> V; | LT2 | 0 | 0 | 0 | 0 |
| E | STMUK_2436 | one-strain | G -> R; A -> A; | SL1344 | 0 | 0 | 0 | 0 |
| E | STMUK_3010 | one-strain | F -> L; | D23580 | 0 | 0 | 0 | 0 |
| E | STMUK_3114 | one-strain | R -> S; P -> P; | LT2 | 0 | 0 | 0 | 0 |
| E | STMUK_3516 | one-strain | K -> R; | LT2 | 0 | 0 | 0 | 0 |
| E | STMUK_4431 | one-strain | V -> I; | LT2 | 0 | 0 | 0 | 0 |
| E | thrA | one-strain | V -> V; | LT2 | 0 | 0 | 0 | 0 |
| E | trpB | one-strain | S -> F; | SL1344 | 0 | 0 | 0 | 0 |
| E | trpD | one-strain | F -> L; | LT2 | 0 | 0 | 0 | 0 |
| E | trpE | one-strain | Q -> K; | LT2 | 0 | 0 | 0 | 0 |
| E | tyrA | one-strain | W -> R; | D23580 | 0 | 0 | 0 | 0 |
| E | tyrB | one-strain | G -> G; | D23580 | 0 | 0 | 0 | 0 |
| E | tyrP | one-strain | S -> S; | LT2 | 1 | 0 | 0 | 0 |
| E | ybgH | one-strain | A -> E; | SL1344 | 0 | 0 | 0 | 0 |
| E | ybgK | one-strain | R -> R; | LT2 | 0 | 0 | 0 | 0 |
| E | yecC | one-strain | R -> R; | D23580 | 0 | 0 | 0 | 0 |
| E | yehY | one-strain | V -> A; | SL1344 | 0 | 0 | 0 | 0 |
| E | yejA | one-strain | W -> R; | LT2 | 0 | 0 | 0 | 0 |
| E | yejE | one-strain | A -> D; | D23580 | 0 | 0 | 0 | 0 |
| E | yhiP | one-strain | F -> L; | D23580 | 0 | 0 | 0 | 0 |
| E | yhjV | one-strain | I -> T; | D23580 | 0 | 0 | 0 | 0 |
| E | yifK | one-strain | L -> L; | LT2 | 0 | 1 | 0 | 0 |
| E | yjeH | one-strain | L -> L; | D23580 | 0 | 0 | 0 | 0 |
| E | allB | one-strain | V -> V; | 14028S | 0 | 0 | 0 | 0 |
| E | cpdB | one-strain | T -> R; | D23580 | 0 | 0 | 0 | 0 |
| E | deoD | one-strain | G -> G; | D23580 | 0 | 0 | 0 | 0 |
| E | guaA | one-strain | P -> L; | LT2 | 0 | 1 | 0 | 0 |
| E | nrdD | one-strain | T -> A; | D23580 | 0 | 0 | 0 | 0 |
| F | nrdI | one-strain | R -> H; | 14028S | 0 | 0 | 0 | 0 |
| F | nupC | one-strain | F -> L; | D23580 | 0 | 0 | 0 | 0 |
| F | purA | one-strain | M -> L; | D23580 | 0 | 1 | 0 | 0 |
| F | purD | one-strain | A -> T; | SL1344 | 0 | 1 | 0 | 0 |
| F | purE | one-strain | Q -> E; | D23580 | 0 | 0 | 0 | 0 |
| F | purG | one-strain | G -> G; | D23580 | 0 | 1 | 0 | 0 |
| F | pyrE | one-strain | T -> T; | SL1344 | 0 | 0 | 0 | 0 |
| F | STMUK_0034 | one-strain | L -> L; | SL1344 | 0 | 0 | 0 | 0 |
| F | STMUK_4090 | one-strain | A -> A; | LT2 | 0 | 0 | 0 | 0 |
| F | STMUK_4398 | one-strain | H -> R; | LT2 | 1 | 0 | 0 | 0 |
| F | ushA | one-strain | S -> Y; G -> V; | LT2 | 0 | 0 | 0 | 0 |
| F | ybbY | one-strain | R -> C; | SL1344 | 0 | 0 | 0 | 0 |
| F | yggV | one-strain | S -> R; | D23580 | 0 | 0 | 0 | 0 |
| F | yicE | one-strain | S -> S; | D23580 | 0 | 0 | 0 | 0 |
| F | araJ | one-strain | G -> G; | D23580 | 0 | 0 | 0 | 0 |
| F | celB | one-strain | M -> I; | D23580 | 0 | 0 | 0 | 0 |
| F | celF | one-strain | A -> E; | D23580 | 0 | 0 | 0 | 0 |
| F | citE2 | one-strain | I -> M; | LT2 | 0 | 0 | 0 | 0 |
| F | emrB | one-strain | T -> N; | D23580 | 0 | 0 | 0 | 0 |
| G | fba | one-strain | E -> G; | D23580 | 0 | 0 | 0 | 0 |
| G | fucK | one-strain | L -> L; | D23580 | 0 | 0 | 0 | 0 |
| G | glgX | one-strain | P -> P; | D23580 | 0 | 0 | 0 | 0 |
| G | glk | one-strain | A -> A; | D23580 | 0 | 0 | 0 | 0 |
| G | glxK | one-strain | V -> V; | LT2 | 0 | 0 | 0 | 0 |
| G | gntT | one-strain | L -> L; | LT2 | 0 | 0 | 0 | 0 |
| G | gsk | one-strain | P -> S; | 14028S | 0 | 0 | 0 | 0 |
| G | idnT | one-strain | L -> L; | LT2 | 0 | 0 | 0 | 0 |
| G | lyxK | one-strain | H -> R; | SL1344 | 0 | 0 | 0 | 0 |
| G | malP | one-strain | R -> R; | D23580 | 0 | 0 | 0 | 0 |
| G | malS | one-strain | Q -> Q; | LT2 | 0 | 0 | 0 | 0 |
| G | melB | one-strain | P -> S; I -> V; | D23580 | 0 | 0 | 0 | 0 |
| G | mglA | one-strain | Q -> H; | SL1344 | 0 | 0 | 0 | 0 |
| G | otsB | one-strain | P -> L; | SL1344 | 0 | 0 | 0 | 0 |
| G | pgtP | one-strain | E -> E; | LT2 | 0 | 0 | 0 | 0 |
| G | pmgI | one-strain | Q -> Q; | D23580 | 0 | 1 | 0 | 0 |
| G | prpB | one-strain | P -> P; | LT2 | 0 | 0 | 0 | 0 |
| G | ptsG | one-strain | I -> I; | D23580 | 0 | 1 | 0 | 0 |
| G | srlE | one-strain | P -> A; | D23580 | 0 | 0 | 0 | 0 |
| G | STMUK_0214 | one-strain | R -> L; | SL1344 | 0 | 0 | 0 | 0 |
| G | STMUK_0579 | one-strain | A -> S; | D23580 | 0 | 0 | 0 | 0 |
| G | STMUK_0581 | one-strain | V -> E; | SL1344 | 0 | 0 | 0 | 0 |
| G | STMUK_0874 | one-strain | S -> F; | SL1344 | 0 | 0 | 0 | 0 |
| G | STMUK_1527 | one-strain | Y -> H; | SL1344 | 0 | 0 | 0 | 0 |
| G | STMUK_1529 | one-strain | E -> E; | D23580 | 0 | 0 | 0 | 0 |
| G | STMUK_1816 | one-strain | T -> I; | LT2 | 0 | 0 | 0 | 0 |
| G | STMUK_2310 | one-strain | M -> I; | D23580 | 0 | 0 | 0 | 0 |
| G | STMUK_2371 | one-strain | L -> F; | 14028S | 0 | 0 | 0 | 0 |
| G | STMUK_2402 | one-strain | I -> N; | SL1344 | 0 | 0 | 0 | 0 |
| G | STMUK_2602 | one-strain | A -> E; | D23580 | 0 | 0 | 0 | 0 |
| G | STMUK_2606 | one-strain | L -> F; | LT2 | 0 | 0 | 0 | 0 |
| G | STMUK_2801 | one-strain | T -> T; | D23580 | 0 | 0 | 0 | 0 |
| G | STMUK_2902 | one-strain | D -> E; L -> L; G -> G; | LT2 | 0 | 0 | 0 | 0 |
| G | STMUK_2948 | one-strain | E -> K; | D23580 | 0 | 0 | 0 | 0 |
| G | STMUK_3108 | one-strain | A -> E; | SL1344 | 0 | 0 | 0 | 1 |
| G | STMUK_3684 | one-strain | V -> A; | D23580 | 0 | 1 | 0 | 0 |
| G | STMUK_3756 | one-strain | S -> Y; | D23580 | 0 | 0 | 0 | 0 |
| G | STMUK_3768 | one-strain | A -> V; E -> D; F -> L; | D23580 | 0 | 0 | 0 | 0 |
| G | STMUK_4404 | one-strain | C -> C; R -> K; | D23580 | 0 | 0 | 0 | 0 |
| G | STMUK_4525 | one-strain | L -> L; | D23580 | 0 | 0 | 0 | 0 |
| G | treC | one-strain | D -> D; | LT2 | 0 | 0 | 0 | 0 |
| G | treF | one-strain | F -> F; | LT2 | 0 | 0 | 0 | 0 |
| G | uhpT | one-strain | G -> G; | D23580 | 0 | 1 | 0 | 0 |
| G | ybhE | one-strain | V -> M; | SL1344 | 0 | 0 | 0 | 0 |
| G | ycaD | one-strain | T -> T; | SL1344 | 0 | 0 | 0 | 0 |
| G | yceE | one-strain | A -> V; | D23580 | 0 | 0 | 0 | 0 |
| G | yceL | one-strain | G -> D; | LT2 | 0 | 0 | 0 | 0 |
| G | yciM | one-strain | G -> G; | SL1344 | 0 | 0 | 0 | 0 |
| G | ydiN | one-strain | E -> Z; | SL1344 | 0 | 0 | 0 | 0 |
| G | ygbL | one-strain | I -> V; | LT2 | 0 | 0 | 0 | 0 |
| G | ygeD | one-strain | T -> M; | SL1344 | 0 | 1 | 0 | 0 |
| G | yicJ | one-strain | L -> L; | LT2 | 0 | 0 | 0 | 0 |
| G | yieO | one-strain | S -> Z; | SL1344 | 0 | 0 | 0 | 0 |
| G | yifZ | one-strain | T -> P; | D23580 | 0 | 0 | 0 | 0 |
| G | yihP | one-strain | A -> S; | D23580 | 0 | 0 | 0 | 0 |
| G | yjiJ | one-strain | A -> T; | 14028S | 0 | 0 | 0 | 0 |
| G | asrB | one-strain | N -> S; | D23580 | 0 | 0 | 0 | 1 |
| G | bioB | one-strain | A -> T; | D23580 | 0 | 0 | 0 | 0 |
| G | bioF | one-strain | A -> T; | SL1344 | 0 | 0 | 0 | 0 |
| G | citG | one-strain | A -> D; | 14028S | 0 | 0 | 0 | 0 |
| G | dxs | one-strain | S -> R; | D23580 | 0 | 0 | 0 | 0 |
| H | folA | one-strain | A -> V; | SL1344 | 0 | 0 | 0 | 0 |
| H | folD | one-strain | A -> T; | SL1344 | 0 | 0 | 0 | 0 |
| H | folP | one-strain | P -> P; | SL1344 | 0 | 0 | 0 | 0 |
| H | ispA | one-strain | P -> P; | D23580 | 0 | 0 | 0 | 0 |
| H | panB | one-strain | A -> V; | D23580 | 0 | 0 | 0 | 0 |
| H | panC | one-strain | A -> T; | D23580 | 0 | 0 | 0 | 0 |
| H | pdxK | one-strain | V -> I; | D23580 | 0 | 0 | 0 | 0 |
| H | pgtC | one-strain | P -> P; | D23580 | 0 | 0 | 0 | 0 |
| H | phnS | one-strain | L -> L; | SL1344 | 0 | 0 | 0 | 0 |
| H | ribD | one-strain | E -> E; | D23580 | 0 | 0 | 0 | 0 |
| H | STMUK_0774 | one-strain | K -> R; | D23580 | 0 | 0 | 0 | 0 |
| H | STMUK_1512 | one-strain | Q -> L; | D23580 | 0 | 0 | 0 | 0 |
| H | STMUK_2911 | one-strain | G -> G; | D23580 | 0 | 0 | 0 | 0 |
| H | thiH | one-strain | P -> P; | SL1344 | 0 | 0 | 0 | 0 |
| H | ubiH | one-strain | A -> R; | LT2 | 0 | 0 | 0 | 0 |
| H | ycjG | one-strain | A -> V; | D23580 | 0 | 0 | 0 | 0 |
| H | ygcY | one-strain | P -> T; | D23580 | 0 | 0 | 0 | 0 |
| H | yggW | one-strain | C -> R; | LT2 | 0 | 0 | 0 | 0 |
| H | yigC | one-strain | L -> L; | D23580 | 1 | 0 | 0 | 0 |
| H | accD | one-strain | A -> A; | D23580 | 0 | 0 | 0 | 0 |
| H | acs | one-strain | D -> D; | SL1344 | 0 | 0 | 0 | 0 |
| H | aes | one-strain | L -> Q; | D23580 | 0 | 0 | 0 | 0 |
| H | cdsA | one-strain | L -> L; | D23580 | 0 | 0 | 0 | 0 |
| H | fabA | one-strain | R -> R; | LT2 | 0 | 0 | 0 | 0 |
| H | fadB | one-strain | L -> L; | D23580 | 1 | 0 | 0 | 0 |
| I | fadI | one-strain | S -> T; | LT2 | 0 | 0 | 0 | 0 |
| I | fadL | one-strain | A -> A; | LT2 | 0 | 0 | 0 | 0 |
| I | glxR | one-strain | S -> S; | LT2 | 0 | 0 | 0 | 0 |
| I | oafA | one-strain | N -> N; | D23580 | 0 | 0 | 0 | 0 |
| I | pldB | one-strain | L -> L; | SL1344 | 0 | 0 | 0 | 0 |
| I | plsB | one-strain | G -> R; | LT2 | 0 | 0 | 0 | 0 |
| I | prpE | one-strain | R -> R; | 14028S | 0 | 0 | 0 | 0 |
| I | sbmA | one-strain | A -> T; | D23580 | 0 | 0 | 0 | 0 |
| I | STMUK_1592 | one-strain | G -> S; | D23580 | 0 | 0 | 0 | 0 |
| I | ybhO | one-strain | A -> E; | SL1344 | 0 | 0 | 0 | 0 |
| I | yfjG | one-strain | G -> Z; | D23580 | 0 | 0 | 0 | 0 |
| I | yihU | one-strain | V -> I; | LT2 | 0 | 0 | 0 | 0 |
| I | yjfJ | one-strain | Q -> Z; | SL1344 | 0 | 0 | 0 | 0 |
| I | cafA | one-strain | D -> D; | LT2 | 0 | 0 | 0 | 0 |
| I | glnS | one-strain | N -> D; | SL1344 | 0 | 0 | 0 | 0 |
| I | glyS | one-strain | V -> V; | LT2 | 0 | 0 | 0 | 0 |
| I | ileS | one-strain | G -> S; | SL1344 | 0 | 0 | 0 | 0 |
| I | ksgA | one-strain | A -> E; | LT2 | 0 | 0 | 0 | 0 |
| I | lasT | one-strain | P -> H; | LT2 | 0 | 0 | 0 | 0 |
| J | map | one-strain | D -> D; | D23580 | 0 | 1 | 0 | 0 |
| J | metG | one-strain | R -> R; | SL1344 | 0 | 0 | 0 | 0 |
| J | miaA | one-strain | L -> L; | LT2 | 0 | 1 | 0 | 0 |
| J | pheT | one-strain | G -> E; | SL1344 | 0 | 0 | 0 | 0 |
| J | pnp | one-strain | V -> A; | D23580 | 0 | 0 | 0 | 0 |
| J | prfC | one-strain | A -> A; | SL1344 | 0 | 0 | 0 | 0 |
| J | proS | one-strain | L -> V; | D23580 | 0 | 0 | 0 | 0 |
| J | queA | one-strain | P -> P; | 14028S | 0 | 0 | 0 | 0 |
| J | rimO | one-strain | G -> A; | 14028S | 0 | 0 | 0 | 0 |
| J | rplC | one-strain | G -> G; | LT2 | 0 | 0 | 0 | 0 |
| J | rpsE | one-strain | R -> R; | 14028S | 0 | 0 | 0 | 0 |
| J | rpsL | one-strain | Y -> Y; | D23580 | 0 | 0 | 0 | 0 |
| J | rpsR | one-strain | A -> R; | LT2 | 0 | 0 | 0 | 0 |
| J | selB | one-strain | A -> V; | D23580 | 0 | 0 | 0 | 0 |
| J | sun | one-strain | R -> R; | D23580 | 0 | 1 | 0 | 0 |
| J | truB | one-strain | K -> K; | SL1344 | 0 | 1 | 0 | 0 |
| J | tyrS | one-strain | A -> V; | D23580 | 0 | 0 | 0 | 0 |
| J | yadB | one-strain | R -> H; | SL1344 | 0 | 0 | 0 | 0 |
| J | yfbG | one-strain | L -> L; | 14028S | 0 | 0 | 0 | 0 |
| J | ygjO | one-strain | A -> T; | D23580 | 0 | 0 | 0 | 0 |
| J | cadC | one-strain | D -> A; | D23580 | 1 | 0 | 0 | 0 |
| J | cspD | one-strain | G -> G; | D23580 | 0 | 0 | 0 | 0 |
| J | cytR | one-strain | V -> E; | 14028S | 0 | 2 | 0 | 0 |
| J | emrR | one-strain | T -> T; | SL1344 | 0 | 0 | 0 | 0 |
| J | envR | one-strain | D -> E; N -> S; | D23580 | 0 | 0 | 0 | 0 |
| J | fhlA | one-strain | A -> D; | LT2 | 0 | 0 | 0 | 0 |
| K | gcvA | one-strain | E -> E; | D23580 | 0 | 0 | 0 | 0 |
| K | melR | one-strain | F -> L; | D23580 | 0 | 0 | 0 | 0 |
| K | metR | one-strain | G -> G; | 14028S | 0 | 0 | 0 | 0 |
| K | mlc | one-strain | P -> S; | SL1344 | 0 | 0 | 0 | 0 |
| K | mtlR | one-strain | R -> R; | LT2 | 0 | 0 | 0 | 1 |
| K | nlp | one-strain | K -> K; | SL1344 | 0 | 0 | 0 | 0 |
| K | orf242 | one-strain | N -> K; A -> T; | LT2 | 0 | 0 | 0 | 0 |
| K | rcsA | one-strain | K -> Q; | SL1344 | 0 | 0 | 0 | 0 |
| K | rhaR | one-strain | G -> S; | SL1344 | 0 | 0 | 0 | 0 |
| K | rpoA | one-strain | I -> I; | D23580 | 0 | 0 | 0 | 0 |
| K | rpoS | one-strain | M -> L; | LT2 | 0 | 1 | 0 | 1 |
| K | slyA | one-strain | E -> D; P -> A; | LT2 | 0 | 0 | 0 | 0 |
| K | STMUK_0031 | one-strain | G -> D; | LT2 | 1 | 0 | 1 | 0 |
| K | STMUK_0032 | one-strain | N -> K; | LT2 | 0 | 0 | 0 | 0 |
| K | STMUK_0657 | one-strain | G -> G; | D23580 | 0 | 0 | 0 | 0 |
| K | STMUK_0697 | one-strain | G -> E; | SL1344 | 0 | 0 | 0 | 0 |
| K | STMUK_1642 | one-strain | H -> Y; D -> D; | D23580 | 0 | 0 | 0 | 0 |
| K | STMUK_2225 | one-strain | D -> D; | LT2 | 0 | 0 | 0 | 0 |
| K | STMUK_3008 | one-strain | M -> I; | LT2 | 0 | 0 | 0 | 0 |
| K | STMUK_3109 | one-strain | V -> I; | D23580 | 0 | 0 | 0 | 1 |
| K | STMUK_3164 | one-strain | T -> I; | D23580 | 0 | 0 | 0 | 0 |
| K | STMUK_3664 | one-strain | A -> S; | D23580 | 0 | 0 | 0 | 0 |
| K | STMUK_4403 | one-strain | E -> E; | D23580 | 1 | 0 | 0 | 1 |
| K | xapR | one-strain | A -> A; | D23580 | 0 | 0 | 0 | 0 |
| K | yafC | one-strain | R -> Q; | D23580 | 0 | 0 | 0 | 0 |
| K | yajF | one-strain | L -> F; | SL1344 | 0 | 0 | 0 | 0 |
| K | ybaO | one-strain | D -> G; | SL1344 | 0 | 0 | 0 | 0 |
| K | ybdO | one-strain | S -> L; | D23580 | 1 | 0 | 0 | 0 |
| K | ydcR | one-strain | A -> A; | D23580 | 0 | 0 | 0 | 0 |
| K | ydeW | one-strain | T -> T; | D23580 | 0 | 0 | 0 | 0 |
| K | ydhB | one-strain | L -> L; | D23580 | 0 | 0 | 0 | 0 |
| K | ydiP | one-strain | I -> I; | SL1344 | 0 | 0 | 0 | 0 |
| K | ygaA | one-strain | P -> L; | D23580 | 0 | 0 | 0 | 0 |
| K | yhaJ | one-strain | V -> V; F -> L; | D23580 | 0 | 0 | 0 | 0 |
| K | yhhY | one-strain | A -> T; | SL1344 | 0 | 0 | 0 | 0 |
| K | yjaB | one-strain | I -> M; | LT2 | 0 | 0 | 0 | 0 |
| K | yqgE | one-strain | G -> S; | D23580 | 0 | 0 | 0 | 0 |
| K | yqhC | one-strain | N -> N; | SL1344 | 0 | 0 | 0 | 0 |
| K | zntR | one-strain | C -> Y; | SL1344 | 0 | 0 | 0 | 0 |
| K | dam | one-strain | D -> D; | SL1344 | 0 | 1 | 0 | 0 |
| K | dnaA | one-strain | R -> R; V -> V; | D23580 | 0 | 0 | 0 | 0 |
| K | gyrB | one-strain | L -> L; | 14028S | 0 | 0 | 0 | 0 |
| K | holA | one-strain | A -> A; | LT2 | 0 | 0 | 0 | 0 |
| K | hrpA | one-strain | S -> R; | LT2 | 0 | 0 | 0 | 0 |
| K | hsdM | one-strain | R -> H; | LT2 | 0 | 0 | 0 | 0 |
| L | hsdR | one-strain | K -> T; | LT2 | 0 | 0 | 0 | 0 |
| L | mfd | one-strain | L -> L; | SL1344 | 0 | 0 | 0 | 0 |
| L | mutL | one-strain | P -> Q; | LT2 | 0 | 0 | 0 | 0 |
| L | nth | one-strain | R -> H; | SL1344 | 0 | 0 | 0 | 0 |
| L | priA | one-strain | L -> L; | SL1344 | 0 | 0 | 0 | 0 |
| L | radC | one-strain | D -> Y; | D23580 | 0 | 0 | 0 | 0 |
| L | rdgC | one-strain | T -> T; | D23580 | 0 | 0 | 0 | 0 |
| L | recC | one-strain | Q -> H; L -> V; | LT2 | 0 | 0 | 0 | 0 |
| L | recN | one-strain | L -> L; | SL1344 | 0 | 0 | 0 | 0 |
| L | rep | one-strain | L -> L; | D23580 | 0 | 0 | 0 | 0 |
| L | rlmL | one-strain | A -> E; | D23580 | 0 | 0 | 0 | 0 |
| L | sbcD | one-strain | Q -> Q; | LT2 | 0 | 0 | 0 | 0 |
| L | srmB | one-strain | G -> G; | D23580 | 0 | 0 | 0 | 0 |
| L | topA | one-strain | E -> E; | D23580 | 0 | 0 | 0 | 0 |
| L | uvrC | one-strain | T -> S; | LT2 | 0 | 0 | 0 | 0 |
| L | wcaH | one-strain | Q -> Q; D -> G; | D23580 | 0 | 0 | 0 | 0 |
| L | ybaZ | one-strain | R -> C; | SL1344 | 0 | 0 | 0 | 0 |
| L | ybjD | one-strain | S -> T; | LT2 | 0 | 0 | 0 | 0 |
| L | ycaJ | one-strain | A -> A; | LT2 | 0 | 0 | 0 | 0 |
| L | ycfH | one-strain | P -> P; | D23580 | 0 | 0 | 0 | 0 |
| L | yicF | one-strain | A -> V; | LT2 | 0 | 0 | 0 | 0 |
| L | aefA | one-strain | L -> L; T -> T; | D23580 | 0 | 1 | 0 | 0 |
| L | ampD | one-strain | A -> T; | LT2 | 0 | 0 | 0 | 0 |
| L | blc | one-strain | S -> G; | LT2 | 1 | 0 | 0 | 0 |
| L | caiT | one-strain | S -> N; | LT2 | 0 | 1 | 0 | 0 |
| L | dacB | one-strain | V -> A; | SL1344 | 0 | 0 | 0 | 0 |
| L | ftsI | one-strain | R -> R; | 14028S | 0 | 0 | 0 | 0 |
| M | gcpE | one-strain | E -> G; | SL1344 | 0 | 0 | 0 | 0 |
| M | imp | one-strain | N -> Y; | LT2 | 0 | 0 | 0 | 0 |
| M | lnt | one-strain | T -> N; | D23580 | 0 | 0 | 0 | 0 |
| M | mraW | one-strain | V -> V; | D23580 | 0 | 0 | 0 | 0 |
| M | mrcA | one-strain | K -> K; | SL1344 | 0 | 0 | 0 | 0 |
| M | mreC | one-strain | D -> N; | SL1344 | 0 | 0 | 0 | 0 |
| M | rfaB | one-strain | P -> P; | LT2 | 0 | 1 | 0 | 1 |
| M | rlpA | one-strain | A -> A; | SL1344 | 0 | 0 | 0 | 0 |
| M | STMUK_0294 | one-strain | P -> S; | D23580 | 0 | 0 | 0 | 0 |
| M | STMUK_0296 | one-strain | R -> K; | LT2 | 0 | 0 | 0 | 0 |
| M | STMUK_0516 | one-strain | D -> E; | D23580 | 0 | 0 | 0 | 0 |
| M | STMUK_0577 | one-strain | R -> R; | SL1344 | 0 | 0 | 0 | 0 |
| M | STMUK_0725 | one-strain | W -> Z; | SL1344 | 0 | 0 | 0 | 1 |
| M | STMUK_0731 | one-strain | D -> G; | 14028S | 0 | 0 | 0 | 0 |
| M | STMUK_1506 | one-strain | P -> S; | SL1344 | 0 | 0 | 0 | 0 |
| M | STMUK_2725 | one-strain | A -> P; | SL1344 | 0 | 0 | 0 | 0 |
| M | STMUK_2744 | one-strain | L -> L; | LT2 | 0 | 0 | 0 | 0 |
| M | wcaE | one-strain | Q -> R; | LT2 | 0 | 0 | 0 | 0 |
| M | wcaI | one-strain | H -> H; | SL1344 | 0 | 0 | 0 | 0 |
| M | yaeT | one-strain | K -> K; | LT2 | 0 | 0 | 0 | 0 |
| M | ybjR | one-strain | G -> S; | LT2 | 0 | 0 | 0 | 0 |
| M | yeeZ | one-strain | P -> H; | D23580 | 0 | 0 | 0 | 0 |
| M | yfhD | one-strain | F -> L; | 14028S | 0 | 0 | 0 | 0 |
| M | yggB | one-strain | E -> D; | D23580 | 0 | 0 | 0 | 0 |
| M | yibD | one-strain | V -> A; | D23580 | 0 | 0 | 1 | 0 |
| M | yohG | one-strain | G -> S; | D23580 | 0 | 0 | 0 | 0 |
| M | bcfB | one-strain | L -> Q; | SL1344 | 1 | 0 | 0 | 0 |
| M | ffh | one-strain | K -> E; | D23580 | 0 | 0 | 0 | 0 |
| M | fimA | one-strain | K -> R; | D23580 | 0 | 0 | 0 | 0 |
| M | flgK | one-strain | A -> A; | SL1344 | 1 | 0 | 0 | 0 |
| M | flhA | one-strain | A -> T; | D23580 | 0 | 0 | 0 | 0 |
| M | flhB | one-strain | N -> I; | SL1344 | 1 | 0 | 0 | 0 |
| N | fliP | one-strain | Y -> Y; | D23580 | 0 | 0 | 0 | 0 |
| N | fljB | one-strain | T -> T; | LT2 | 1 | 0 | 0 | 0 |
| N | hofB | one-strain | R -> C; | LT2 | 0 | 0 | 0 | 0 |
| N | invA | one-strain | R -> R; | SL1344 | 1 | 0 | 0 | 0 |
| N | lepB | one-strain | F -> F; | LT2 | 0 | 0 | 0 | 0 |
| N | lpfC | one-strain | S -> S; | LT2 | 0 | 0 | 0 | 0 |
| N | lpxK | one-strain | L -> L; | SL1344 | 0 | 0 | 0 | 0 |
| N | secD | one-strain | M -> I; L -> L; | LT2 | 0 | 0 | 0 | 0 |
| N | ssaU | one-strain | H -> Y; | D23580 | 1 | 1 | 1 | 1 |
| N | stbC | one-strain | S -> G; | LT2 | 0 | 0 | 0 | 0 |
| N | stbE | one-strain | N -> S; | LT2 | 0 | 0 | 0 | 0 |
| N | stcC | one-strain | R -> H; | D23580 | 1 | 0 | 1 | 0 |
| N | stfC | one-strain | G -> G; R -> L; | D23580 | 0 | 0 | 0 | 0 |
| N | sthB | one-strain | G -> V; | LT2 | 0 | 0 | 0 | 1 |
| N | stiA | one-strain | L -> L; | D23580 | 0 | 0 | 0 | 0 |
| N | stiC | one-strain | N -> S; | D23580 | 0 | 0 | 0 | 0 |
| N | STMUK_1626 | one-strain | I -> R; | LT2 | 0 | 0 | 0 | 0 |
| N | STMUK_3205 | one-strain | S -> I; | SL1344 | 0 | 0 | 0 | 0 |
| N | trg | one-strain | A -> R; | LT2 | 0 | 0 | 0 | 0 |
| N | ahpF | one-strain | I -> I; G -> G; | LT2 | 0 | 0 | 0 | 0 |
| N | bcfH | one-strain | L -> Q; | D23580 | 0 | 0 | 0 | 0 |
| N | clpB | one-strain | G -> S; | D23580 | 0 | 0 | 0 | 0 |
| N | cypD | one-strain | E -> E; | LT2 | 0 | 0 | 0 | 0 |
| N | dnaJ | one-strain | A -> A; | D23580 | 0 | 0 | 0 | 0 |
| N | dnaK | one-strain | A -> A; | SL1344 | 0 | 0 | 0 | 0 |
| O | dsbG | one-strain | D -> N; | SL1344 | 0 | 0 | 0 | 0 |
| O | hflK | one-strain | S -> S; | D23580 | 0 | 0 | 0 | 0 |
| O | hslO | one-strain | A -> T; | SL1344 | 0 | 0 | 0 | 0 |
| O | lon | one-strain | L -> L; | D23580 | 0 | 0 | 0 | 0 |
| O | lonH | one-strain | A -> V; | LT2 | 0 | 0 | 0 | 0 |
| O | nrdH | one-strain | T -> T; | SL1344 | 0 | 0 | 0 | 0 |
| O | radA | one-strain | E -> E; | D23580 | 0 | 0 | 0 | 0 |
| O | STMUK_0274 | one-strain | V -> V; | SL1344 | 0 | 0 | 0 | 0 |
| O | STMUK_1635 | one-strain | T -> M; | LT2 | 1 | 0 | 0 | 0 |
| O | STMUK_4052 | one-strain | A -> T; | D23580 | 0 | 0 | 0 | 0 |
| O | surA | one-strain | G -> S; | D23580 | 0 | 0 | 0 | 0 |
| O | trxC | one-strain | V -> V; | LT2 | 0 | 0 | 0 | 0 |
| O | ybbN | one-strain | N -> N; | SL1344 | 0 | 0 | 0 | 0 |
| O | yegD | one-strain | G -> S; | D23580 | 0 | 0 | 0 | 0 |
| O | ygcF | one-strain | R -> R; | LT2 | 0 | 0 | 0 | 0 |
| O | yibF | one-strain | S -> S; | LT2 | 0 | 0 | 0 | 0 |
| O | yifB | one-strain | R -> H; | SL1344 | 0 | 0 | 0 | 0 |
| O | yliJ | one-strain | T -> T; | D23580 | 0 | 0 | 0 | 0 |
| O | btuD | one-strain | L -> F; | SL1344 | 0 | 0 | 0 | 0 |
| O | cbiN | one-strain | E -> G; | D23580 | 0 | 0 | 0 | 0 |
| O | cbiO | one-strain | I -> I; | LT2 | 0 | 0 | 0 | 0 |
| O | cutC | one-strain | D -> E; | D23580 | 0 | 0 | 0 | 0 |
| O | cyaY | one-strain | K -> N; | 14028S | 0 | 0 | 0 | 0 |
| O | cysI | one-strain | K -> K; | SL1344 | 0 | 0 | 0 | 0 |
| P | fepC | one-strain | S -> G; | D23580 | 0 | 0 | 0 | 0 |
| P | fhuB | one-strain | M -> T; | LT2 | 0 | 0 | 0 | 0 |
| P | fhuC | one-strain | E -> E; | D23580 | 0 | 0 | 0 | 0 |
| P | fhuE | one-strain | D -> E; A -> T; | 14028S | 0 | 0 | 0 | 0 |
| P | foxA | one-strain | L -> P; | LT2 | 0 | 0 | 0 | 0 |
| P | ftnB | one-strain | N -> S; | SL1344 | 0 | 0 | 0 | 0 |
| P | katE | one-strain | E -> G; | D23580 | 0 | 0 | 0 | 0 |
| P | katG | one-strain | R -> R; | LT2 | 0 | 0 | 0 | 0 |
| P | kdpA | one-strain | R -> C; | LT2 | 0 | 0 | 0 | 0 |
| P | kdpB | one-strain | V -> A; | SL1344 | 0 | 1 | 0 | 0 |
| P | kefB | one-strain | A -> A; | D23580 | 0 | 0 | 0 | 0 |
| P | kefC | one-strain | R -> R; | SL1344 | 0 | 0 | 0 | 0 |
| P | napD | one-strain | G -> G; | D23580 | 0 | 0 | 0 | 0 |
| P | narH | one-strain | E -> E; | D23580 | 0 | 0 | 0 | 0 |
| P | nrfA | one-strain | T -> T; | D23580 | 0 | 0 | 0 | 0 |
| P | opgD | one-strain | A -> V; | D23580 | 0 | 0 | 0 | 0 |
| P | opgG | one-strain | T -> P; | LT2 | 0 | 0 | 0 | 0 |
| P | pstA | one-strain | D -> D; | D23580 | 0 | 0 | 0 | 0 |
| P | sitA | one-strain | A -> A; | D23580 | 0 | 0 | 0 | 0 |
| P | sodB | one-strain | V -> A; | LT2 | 0 | 0 | 0 | 0 |
| P | sseA | one-strain | F -> F; | SL1344 | 1 | 0 | 1 | 1 |
| P | STMUK_0771 | one-strain | P -> T; | LT2 | 0 | 0 | 0 | 0 |
| P | STMUK_2435 | one-strain | S -> A; | SL1344 | 0 | 0 | 0 | 0 |
| P | yadQ | one-strain | A -> E; | LT2 | 0 | 0 | 0 | 0 |
| P | ybaL | one-strain | Q -> Q; | SL1344 | 0 | 0 | 0 | 0 |
| P | yeaN | one-strain | A -> A; | D23580 | 0 | 0 | 0 | 0 |
| P | yfdC | one-strain | G -> S; | LT2 | 0 | 0 | 0 | 0 |
| P | entA | one-strain | S -> S; L -> L; | LT2 | 0 | 0 | 0 | 0 |
| P | entB | one-strain | S -> S; | D23580 | 0 | 0 | 0 | 0 |
| P | entE | one-strain | L -> L; K -> K; | D23580 | 0 | 0 | 0 | 0 |
| P | entF | one-strain | P -> S; | SL1344 | 0 | 0 | 0 | 0 |
| P | STMUK_1575 | one-strain | S -> S; | SL1344 | 0 | 0 | 0 | 0 |
| P | STMUK_2726 | one-strain | P -> P; | D23580 | 0 | 0 | 0 | 0 |
| P | yafE | one-strain | A -> V; | SL1344 | 0 | 0 | 0 | 0 |
| Q | yafS | one-strain | S -> F; | D23580 | 0 | 0 | 1 | 0 |
| Q | ybjY | one-strain | S -> L; | D23580 | 0 | 0 | 0 | 0 |
| Q | ydhE | one-strain | A -> R; | LT2 | 0 | 0 | 0 | 0 |
| Q | yecP | one-strain | G -> G; | SL1344 | 0 | 0 | 0 | 0 |
| Q | yegO | one-strain | Q -> Q; | LT2 | 0 | 0 | 0 | 0 |
| Q | adhA | one-strain | I -> I; | LT2 | 0 | 0 | 0 | 0 |
| Q | cvpA | one-strain | F -> C; | 14028S | 0 | 1 | 0 | 0 |
| Q | dkgB | one-strain | N -> S; | LT2 | 0 | 0 | 0 | 0 |
| Q | elaA | one-strain | V -> I; | D23580 | 1 | 0 | 0 | 0 |
| Q | elaC | one-strain | V -> V; | SL1344 | 0 | 0 | 0 | 0 |
| Q | hflX | one-strain | A -> A; | LT2 | 0 | 0 | 0 | 0 |
| Q | modE | one-strain | G -> S; | D23580 | 0 | 0 | 0 | 0 |
| R | mviM | one-strain | G -> S; | LT2 | 1 | 0 | 0 | 0 |
| R | sfbA | one-strain | N -> N; | SL1344 | 0 | 0 | 0 | 0 |
| R | sscA | one-strain | T -> A; | D23580 | 0 | 1 | 0 | 1 |
| R | sspB | one-strain | P -> L; | D23580 | 0 | 0 | 0 | 0 |
| R | STMUK_0444 | one-strain | N -> S; | D23580 | 0 | 0 | 0 | 0 |
| R | STMUK_0445 | one-strain | M -> I; | D23580 | 0 | 0 | 0 | 0 |
| R | STMUK_1732 | one-strain | E -> V; | D23580 | 0 | 0 | 0 | 1 |
| R | STMUK_2939 | one-strain | I -> S; | LT2 | 0 | 0 | 0 | 0 |
| R | STMUK_3584 | one-strain | V -> A; | D23580 | 0 | 0 | 0 | 0 |
| R | STMUK_4292 | one-strain | A -> V; | D23580 | 0 | 0 | 0 | 0 |
| R | STMUK_4418 | one-strain | P -> S; | D23580 | 0 | 0 | 0 | 0 |
| R | sufB | one-strain | S -> P; | D23580 | 0 | 0 | 0 | 0 |
| R | tldD | one-strain | N -> N; | LT2 | 0 | 0 | 0 | 0 |
| R | uup | one-strain | L -> L; | LT2 | 0 | 0 | 0 | 0 |
| R | wzxC | one-strain | L -> P; | SL1344 | 0 | 0 | 0 | 0 |
| R | yadH | one-strain | G -> V; | LT2 | 0 | 0 | 0 | 0 |
| R | yaeE | one-strain | I -> T; | 14028S | 1 | 0 | 0 | 0 |
| R | ybhP | one-strain | H -> H; | D23580 | 0 | 0 | 0 | 0 |
| R | ybhR | one-strain | L -> L; | 14028S | 0 | 0 | 0 | 0 |
| R | ybiT | one-strain | T -> M; | SL1344 | 0 | 0 | 0 | 0 |
| R | ybiV1 | one-strain | L -> F; | D23580 | 0 | 0 | 0 | 0 |
| R | ybiV2 | one-strain | N -> K; | 14028S | 0 | 0 | 0 | 0 |
| R | ycaI | one-strain | N -> N; | SL1344 | 0 | 0 | 0 | 0 |
| R | ychF | one-strain | V -> A; | LT2 | 0 | 1 | 0 | 0 |
| R | yedP | one-strain | T -> T; | SL1344 | 0 | 0 | 0 | 0 |
| R | yejM | one-strain | G -> G; | SL1344 | 0 | 0 | 0 | 0 |
| R | yfeH | one-strain | K -> N; L -> V; | LT2 | 0 | 0 | 0 | 0 |
| R | yfeU | one-strain | R -> R; | D23580 | 0 | 0 | 0 | 0 |
| R | yfgB | one-strain | L -> L; | LT2 | 0 | 0 | 0 | 0 |
| R | ygaD | one-strain | A -> V; | D23580 | 0 | 0 | 0 | 0 |
| R | ygaF | one-strain | P -> S; | SL1344 | 0 | 0 | 0 | 0 |
| R | ygcB | one-strain | D -> D; | D23580 | 0 | 0 | 0 | 0 |
| R | ygdE | one-strain | M -> I; | D23580 | 0 | 0 | 0 | 0 |
| R | ygdH | one-strain | V -> V; | LT2 | 0 | 0 | 0 | 0 |
| R | yggA | one-strain | G -> E; | D23580 | 0 | 0 | 0 | 0 |
| R | ygjR | one-strain | L -> L; | LT2 | 0 | 0 | 0 | 0 |
| R | yhbO | one-strain | F -> V; | D23580 | 0 | 0 | 0 | 0 |
| R | yheT | one-strain | N -> D; | LT2 | 0 | 0 | 0 | 0 |
| R | yhiN | one-strain | A -> A; | D23580 | 0 | 0 | 0 | 0 |
| R | yidE | one-strain | W -> C; | SL1344 | 0 | 0 | 0 | 0 |
| R | yijP | one-strain | R -> R; | SL1344 | 0 | 0 | 0 | 0 |
| R | yjdB | one-strain | L -> P; | D23580 | 0 | 0 | 1 | 0 |
| R | yjeQ | one-strain | E -> K; | SL1344 | 0 | 0 | 0 | 0 |
| R | yjgP | one-strain | V -> V; | D23580 | 0 | 0 | 0 | 0 |
| R | yjjK | one-strain | V -> A; | D23580 | 0 | 0 | 0 | 0 |
| R | yqiA | one-strain | D -> Y; | LT2 | 0 | 0 | 0 | 0 |
| R | yraM | one-strain | I -> I; | SL1344 | 0 | 0 | 0 | 0 |
| R | yrbI | one-strain | A -> P; | SL1344 | 0 | 0 | 0 | 0 |
| R | gcd | one-strain | P -> L; | 14028S | 0 | 0 | 0 | 0 |
| R | pqiA | one-strain | V -> I; | SL1344 | 0 | 0 | 0 | 0 |
| R | rtcB | one-strain | I -> S; | LT2 | 0 | 0 | 0 | 0 |
| R | sopA | one-strain | T -> A; | D23580 | 0 | 0 | 0 | 0 |
| R | STMUK_0164 | one-strain | A -> A; | LT2 | 0 | 0 | 0 | 0 |
| R | STMUK_0285 | one-strain | L -> L; | D23580 | 0 | 0 | 0 | 0 |
| R | STMUK_0292 | one-strain | T -> S; | LT2 | 0 | 0 | 0 | 0 |
| S | STMUK_0387 | one-strain | P -> P; | SL1344 | 0 | 0 | 0 | 0 |
| S | STMUK_0571 | one-strain | S -> S; V -> A; | D23580 | 0 | 0 | 0 | 0 |
| S | STMUK_1098 | one-strain | H -> Y; | D23580 | 0 | 0 | 0 | 0 |
| S | STMUK_1590 | one-strain | A -> V; | D23580 | 1 | 0 | 0 | 0 |
| S | STMUK_2776 | one-strain | L -> L; | SL1344 | 0 | 0 | 0 | 0 |
| S | STMUK_3159 | one-strain | L -> L; | LT2 | 0 | 0 | 0 | 0 |
| S | STMUK_3261 | one-strain | L -> L; | D23580 | 0 | 0 | 0 | 0 |
| S | STMUK_3364 | one-strain | P -> P; | D23580 | 0 | 0 | 0 | 0 |
| S | STMUK_3534 | one-strain | T -> A; | LT2 | 0 | 0 | 0 | 0 |
| S | virK | one-strain | R -> R; | LT2 | 0 | 0 | 0 | 0 |
| S | yaaA | one-strain | K -> K; | D23580 | 0 | 0 | 0 | 0 |
| S | yabI | one-strain | D -> G; | LT2 | 0 | 0 | 0 | 0 |
| S | ybaY | one-strain | S -> S; | D23580 | 0 | 0 | 0 | 0 |
| S | ybgI | one-strain | D -> D; | D23580 | 0 | 0 | 0 | 0 |
| S | ycaQ | one-strain | P -> S; | D23580 | 0 | 0 | 0 | 0 |
| S | ycbC | one-strain | T -> T; | LT2 | 0 | 0 | 0 | 0 |
| S | yceI | one-strain | Y -> Y; | SL1344 | 0 | 0 | 0 | 0 |
| S | ycfS | one-strain | P -> P; | SL1344 | 0 | 0 | 0 | 0 |
| S | ydaL | one-strain | P -> P; | LT2 | 0 | 0 | 0 | 0 |
| S | ydiU | one-strain | T -> I; | SL1344 | 0 | 0 | 0 | 0 |
| S | yeiH | one-strain | R -> C; | LT2 | 0 | 0 | 0 | 0 |
| S | yfjF | one-strain | P -> P; | D23580 | 0 | 0 | 0 | 0 |
| S | yghB | one-strain | L -> L; | D23580 | 0 | 0 | 0 | 0 |
| S | ygiK | one-strain | M -> I; | LT2 | 0 | 0 | 0 | 0 |
| S | yicC | one-strain | E -> E; | D23580 | 0 | 0 | 0 | 0 |
| S | yigA | one-strain | L -> F; | D23580 | 0 | 0 | 0 | 0 |
| S | yigZ | one-strain | A -> V; | D23580 | 0 | 0 | 0 | 0 |
| S | yjaG | one-strain | E -> E; | SL1344 | 0 | 0 | 1 | 0 |
| S | yjjX | one-strain | I -> T; | D23580 | 0 | 0 | 0 | 0 |
| S | ylbA | one-strain | A -> A; | LT2 | 0 | 0 | 0 | 0 |
| S | yqcC | one-strain | E -> E; | LT2 | 0 | 0 | 0 | 0 |
| S | yqjF | one-strain | P -> Q; | 14028S | 0 | 0 | 0 | 0 |
| S | ytfP | one-strain | N -> D; | 14028S | 0 | 0 | 0 | 0 |
| S | arcB | one-strain | A -> V; | LT2 | 0 | 0 | 0 | 0 |
| S | cpxA | one-strain | L -> L; | LT2 | 0 | 0 | 0 | 0 |
| S | creC | one-strain | V -> I; | LT2 | 0 | 0 | 0 | 0 |
| S | kdpD | one-strain | P -> P; | SL1344 | 0 | 1 | 0 | 0 |
| S | pgtB | one-strain | L -> L; | SL1344 | 0 | 0 | 0 | 0 |
| S | ptsP | one-strain | L -> L; | LT2 | 0 | 0 | 0 | 0 |
| S | rcsB | one-strain | S -> S; | LT2 | 0 | 0 | 0 | 0 |
| T | smp | one-strain | Q -> H; L -> V; | LT2 | 0 | 0 | 0 | 0 |
| T | ssrA | one-strain | D -> G; L -> L; | D23580 | 1 | 1 | 1 | 0 |
| T | STMUK_0054 | one-strain | V -> A; | LT2 | 0 | 0 | 0 | 0 |
| T | STMUK_0576 | one-strain | A -> T; | SL1344 | 0 | 0 | 0 | 0 |
| T | STMUK_1965 | one-strain | T -> R; | D23580 | 0 | 0 | 0 | 0 |
| T | STMUK_4521 | one-strain | S -> G; | D23580 | 0 | 0 | 0 | 0 |
| T | ttrS | one-strain | V -> G; | D23580 | 0 | 0 | 0 | 0 |
| T | ydiV | one-strain | E -> K; | SL1344 | 0 | 0 | 0 | 0 |
| T | yfhA | one-strain | N -> N; | D23580 | 0 | 0 | 0 | 0 |
| T | ygiY | one-strain | I -> I; | SL1344 | 0 | 1 | 0 | 0 |
| T | ylaB | one-strain | S -> N; | SL1344 | 0 | 0 | 0 | 0 |
| T | yojN | one-strain | T -> T; | SL1344 | 0 | 0 | 0 | 0 |
| T | ttrC | one-strain | A -> A; H -> R; | LT2 | 0 | 0 | 0 | 0 |
| T | tus | one-strain | N -> S; | D23580 | 0 | 0 | 0 | 0 |
| T | wecF | one-strain | E -> E; | SL1344 | 0 | 0 | 0 | 0 |
| T | yacF | one-strain | D -> G; | SL1344 | 0 | 0 | 0 | 0 |
| T | yajR | one-strain | Q -> L; | D23580 | 0 | 0 | 0 | 0 |
| T | ybeS | one-strain | A -> A; | LT2 | 0 | 0 | 0 | 0 |
| T | ybfE | one-strain | Z -> Q; | LT2 | 0 | 0 | 0 | 0 |
| - | ycdZ | one-strain | V -> G; | LT2 | 0 | 0 | 0 | 0 |
| - | yciW | one-strain | W -> Z; | LT2 | 0 | 0 | 0 | 0 |
| - | ycjE | one-strain | G -> G; | SL1344 | 0 | 0 | 0 | 0 |
| - | ydbH | one-strain | G -> G; | 14028S | 0 | 0 | 0 | 0 |
| - | ydgJ | one-strain | A -> R; | LT2 | 0 | 0 | 0 | 0 |
| - | ydiF | one-strain | E -> G; | LT2 | 0 | 0 | 0 | 0 |
| - | yeaR | one-strain | G -> D; | LT2 | 0 | 0 | 0 | 0 |
| - | yehR | one-strain | A -> T; | D23580 | 0 | 0 | 0 | 0 |
| - | yeiP | one-strain | T -> T; | 14028S | 0 | 0 | 0 | 0 |
| - | yfeD | one-strain | F -> S; | D23580 | 0 | 0 | 0 | 0 |
| - | yfeK | one-strain | K -> N; L -> V; | LT2 | 0 | 0 | 0 | 0 |
| - | yhaN | one-strain | R -> R; | SL1344 | 0 | 0 | 0 | 0 |
| - | yhcG | one-strain | A -> S; | D23580 | 0 | 0 | 0 | 0 |
| - | yhfL | one-strain | V -> V; | SL1344 | 0 | 0 | 0 | 0 |
| - | yhjS | one-strain | D -> A; | LT2 | 0 | 0 | 0 | 0 |
| - | yidF | one-strain | A -> A; | LT2 | 0 | 0 | 0 | 0 |
| - | yidR | one-strain | T -> A; | D23580 | 0 | 0 | 0 | 0 |
| - | yigF | one-strain | I -> V; | D23580 | 0 | 0 | 1 | 0 |
| - | yjbF | one-strain | M -> V; | LT2 | 0 | 0 | 0 | 0 |
| - | ypfG | one-strain | A -> V; | LT2 | 0 | 0 | 0 | 0 |

^a^. If the gene carrying SNPs was detected to be potentially virulent by public data, the SNP was marked as 1.
