# Supplementary material for: Comparative Genome Analysis of the High Pathogenicity Salmonella Typhimurium Strain UK-1
Source: PLoS One. 2012 Jul 6;7(7):e40645. doi: 10.1371/journal.pone.0040645 (PMC3391293; doi:10.1371/journal.pone.0040645)
Supplement: Table S4 — Insertions detected in the UK-1 strain by referring to the other four S. Typhimurium strains. (DOC) [file pone.0040645.s006.doc]

Table S4. Insertions detected in the UK-1 strain by referring to the other four *S.* Typhimurium strains.

| **Indel id** | **Location** | **Reference strain** | **Configuration** | **Strand** | **Genes** | **Frame shift** |
| --- | --- | --- | --- | --- | --- | --- |
| INS-01 | 381679..381684 | LT2 | CACTAT | - | *stbC* | NO |
| INS-02 | 997482..997511 | LT2 | GTAGCGCCGCAGCCACAGTATCAGCAGCCG |  | Non-coding region |  |
| INS-03 | 1886489..1886491 | LT2 | GCG | - | *yoaE* | NO |
| INS-04^a^ | 2664774..2664791 | LT2, 14028s, D23580, and SL1344 | CCAGCGCATCCAGCGCAT | - | STMUK_2562 | NO |
| INS-05 | 2740369,  2740446 ..2740529 | LT2 | G,  GGGTTCGTGGGCAGTACACCACGTCTGCTTCCGTCTGGCCGCTGGTGATGGTTTCCCGGTCGGGGAACAACTTCACAAACTGA | - | STMUK_2623 | NO, (3’-end truncated in LT2) |
| INS-06 | 2875046..2875055 | LT2 | TTGCCGCGAT | - | STMUK_2752 | YES |
| INS-07 | 2970191..2970193 | LT2 | GCG | + | *hypD* | NO |
| INS-08 | 3590429..3590431 | LT2 | AAT |  | Non-coding region |  |
| INS-09 | 3591369..3591401 | LT2 | TGTAACCCCGCCCGATGATAGCGGCGATGACGA |  | Non-coding region |  |
| INS-10 | 3619153..3619155 | LT2 | GCC | + | *pckA* | NO |
| INS-11 | 3724014..3724016 | LT2 | CCG | + | *yhiP* | NO |
| INS-12 | 3259717..3259718 | 14028s | TT | - | STMUK_3121 | YES |
| INS-13 | 900936..900939 | D23580 | GACC | - | *ybiP* | YES |
| INS-14 | 967593..967607 | D23580 | GCCGTATACGTCGTC | - | *ltaA* | NO |
| INS-15 | 977787..977788 | D23580 | CA | + | *ybjZ* | YES |
| INS-16 | 1552915..1552925 | D23580 | ATAAAAAAGAG | - | *ydeE* | YES |
| INS-17 | 1557393..1557398 | D23580 | CAGCAG | - | *ydeA* | NO |
| INS-18 | 1581211..1581213 | D23580 | GTA | - | STMUK_1512 | NO |
| INS-19 | 2012814..2012825 | D23580 | CGGCAGCGGCGT | - | *yedD* | NO |
| INS-20 | 3139946..3139949 | D23580 | GATC | + | STMUK_3000 | YES |
| INS-21 | 3710325..3710330 | D23580 | GACGCC | - | *yhhS* | NO |
| INS-22 | 4172111..4172122 | D23580 | AGACGACGAAGA | + | *yihI* | NO |
| INS-23 | 4427426..4427437 | D23580 | TCGCCGACGCGA | - | *Qor* | NO |
| INS-24 | 4776939..4776948 | D23580 | GTAATTCGTC | + | STMUK_4549 | Promoter |
| INS-25 | 4734584..4734595 | D23580 | GCTGGAAAAAGG | + | STMUK_4505 | NO |
| INS-26 | 700489..700566 | SL1344 | CGCACTCACCGGGGCAGATACCGGGGCCGCGGCAGCAGGCGCGCTTACCGGCGTCGCAGTCGCTGGAGCCGTCGCTGG | - | *rlpA* | NO |
| INS-27 | 2978839..2978841 | SL1344 | AAA | - | *avrA* | NO |
| INS-28 | 3027098..3027112 | SL1344 | CGGTATCGCCCGCCG | - | *ygbM* | NO |
| INS-29 | 4012239..4012241 | SL1344 | AAG | + | STMUK_3833 | NO |
| INS-30 | 4060031..4060034 | SL1344 | AGCC | - | *yieO* | YES |
| INS-31 | 4780879..4780881 | SL1344 | GCC | - | *yjjI* | NO |

1. This insertion was only detected in the UK-1 strain.
