# Supplementary material for: Comparative Genome Analysis of the High Pathogenicity Salmonella Typhimurium Strain UK-1
Source: PLoS One. 2012 Jul 6;7(7):e40645. doi: 10.1371/journal.pone.0040645 (PMC3391293; doi:10.1371/journal.pone.0040645)
Supplement: Table S5 — 43 VNTRs identified in the five S. Typhimurium strains. (DOC) [file pone.0040645.s007.doc]

Table S5. 43 VNTRs identified in the five *S.* Typhimurium strains.

| **ID** | **Name** | **Repeat Configuration** | **UK-1 Locus** | **Strains** | | | | |
| --- | --- | --- | --- | --- | --- | --- | --- | --- |
|  |  |  |  | **UK-1** | **LT2** | **14028s** | **D23580** | **SL1344** |
| VNTR-01 ^a^ | STTR1 | [45 bp] | 815518 | 9.1 | 9.1 | 9.1 | 9.1 | 9.1 |
| VNTR-02 ^a^ |  | [155 bp] | 819953 | 2.6 |  | 2.6 | 2.6 | 2.6 |
| VNTR-03 ^ab^ | STTR7 | [39 bp] | 997189 | 7.5 | 8.1 | 7.5 | 7.5 | 7.5 |
| VNTR-04 ^a^ |  | CAGCAGCCGGTAGCGCCGCAGCCACAGTAT | 997444 | 2.3 |  | 2.3 |  |  |
| VNTR-05 ^a^ |  | GAAAACAGGGATAGTTATCCCC | 1064337 | 3.6 | 3.6 | 3.6 |  | 3.6 |
| VNTR-06 ^a^ |  | [184 bp] | 1181777 | 2.4 |  | 2.4 | 2.4 | 2.4 |
| VNTR-07 ^a^ |  | [93 bp] | 1489784 | 5.4 | 5.4 | 5.4 | 5.4 | 5.4 |
| VNTR-08 ^a^ |  | [174 bp] | 1811806 | 4.2 | 4.2 | 4.2 | 4.2 | 4.2 |
| VNTR-09 ^a^ | STTR2 | [60 bp] | 2341682 | 10.3 | 10.3 | 10.3 | 10.3 | 10.3 |
| VNTR-10 ^a^ | 4810628 | [118 bp] | 2531582 | 2.7 | 2.7 | 2.7 | 2.7 | 2.7 |
| VNTR-11 ^a^ | 2628542 | [36 bp] | 2628288 | 4 | 4 | 4 | 4 | 4 |
| VNTR-12 ^a^ | STTR4 | [189 bp] | 2630378 | 5.2 | 5.2 | 5.2 | 5.2 | 5.2 |
| VNTR-13 ^ab^ | STTR6 | GCAAGG | 2730631 | 8.7 | 13.7 | 9.7 | 9.7 | 8.7 |
| VNTR-14 ^a^ |  | [105 bp] | 2768796 | 2.5 |  |  |  |  |
| VNTR-15 ^a^ |  | CTATCCCCGTTTTC[AG]GGGATAA | 2768876 | 7.2 |  |  |  |  |
| VNTR-16 ^a^ |  | [133 bp] | 2938180 | 1.9 | 1.9 | 1.9 | 1.9 | 1.9 |
| VNTR-17 ^a^ |  | [139 bp] | 2938684 | 2.6 | 2.6 | 2.6 | 2.6 | 2.6 |
| VNTR-18 ^a^ |  | [121 bp] | 3045681 | 2.2 |  | 2.2 | 2.2 | 2.2 |
| VNTR-19 ^ab^ | STTR5 or Sal16 | CACGAC | 3151921 | 26.3 | 13.3 | 20.3 | 7.3 | 8.3 |
| VNTR-20 ^a^ | STTR8 | [108 bp] | 3379950 | 6.5 | 6.5 | 6.5 | 6.5 | 6.5 |
| VNTR-21 ^a^ |  | [33 bp] | 3407072 | 2.8 | 2.8 | 2.8 | 2.8 | 2.8 |
| VNTR-22 ^a^ |  | ATGGCGGCAACGTCACCCCGCCCGACG | 3590943 | 3.3 |  | 3.3 | 3.3 | 3.3 |
| VNTR-23 ^ab^ | STTR3 | [33 bp] | 3591011 | 11.9 | 10.9 | 11.9 | 9.9 | 11.9 |
| VNTR-24 ^a^ |  | [42 bp] | 3841901 | 2.3 | 2.3 | 2.3 | 2.3 | 2.3 |
| VNTR-25 ^a^ |  | [42 bp] | 3843640 | 3.3 | 3.3 | 3.3 | 3.3 | 3.3 |
| VNTR-26 ^a^ |  | [272 bp] | 3843724 | 3 | 3 | 3 | 3 | 3 |
| VNTR-27 ^a^ |  | [42 bp] | 3844257 | 2 | 2 | 2 | 2 | 2 |
| VNTR-28 ^a^ |  | [53 bp] | 3917755 | 2.9 |  |  |  |  |
| VNTR-29 ^a^ |  | [169 bp] | 4382218 | 2.9 | 2.9 | 2.9 | 2.9 | 2.9 |
| VNTR-30 ^a^ |  | [232 bp] | 4556856 | 2.3 | 2.3 | 2.3 | 2.3 | 2.3 |
| VNTR-31 ^a^ | 2531837 | [117 bp] | 4771065 | 2.8 | 2.8 | 2.8 | 2.8 | 2.8 |
| VNTR-32 |  | ACAGCAGGA |  |  |  |  | 5.9 |  |
| VNTR-33 |  | TGCGATGTC |  |  | 4.4 |  |  |  |
| VNTR-34 |  | ATCCCCGAAAAGAGGGAAGGGC |  |  |  |  | 3.8 |  |
| VNTR-35 |  | TTACCTGATTCGGGTAAACTTTTC |  |  |  | 3.8 | 3.8 |  |
| VNTR-36 |  | [54 bp] |  |  | 2.4 | 2.4 | 2.4 | 2.4 |
| VNTR-37 |  | [60 bp] |  |  |  | 5.3 |  |  |
| VNTR-38 |  | [61 bp] |  |  |  | 2.5 |  | 2.5 |
| VNTR-39 |  | [61 bp] |  |  | 2.5 |  |  |  |
| VNTR-40 |  | [132 bp] |  |  |  |  | 2.4 |  |
| VNTR-41 | 0819457 | [155 bp] |  |  | 3.6 |  |  |  |
| VNTR-42 |  | [184 bp] |  |  | 3.4 |  |  |  |
| VNTR-43 |  | [200 bp] |  |  | 2 |  |  |  |

1. VNTRs detected in the UK-1 strain.
2. VNTRs identified among the five strains with different copy numbers.
